# The Protective Effect of Phenolic Acids on Liver Disease: A Review of Possible Mechanisms

**DOI:** 10.3390/antiox14101247

**Published:** 2025-10-17

**Authors:** Xinyi Ma, Suhui Xiong, Feng Xiang, Yamei Li, Yan Lin, Yuexin Liu, Limei Lin, Jingchen Xie

**Affiliations:** 1 School of Pharmacy, Hunan University of Chinese Medicine, Changsha 410208, China; mxy123@stu.hnucm.edu.cn (X.M.); 20222058@stu.hnucm.edu.cn (S.X.); 20232050@stu.hnucm.edu.cn (F.X.); yameili@hnucm.edu.cn (Y.L.); linyan198210@163.com (Y.L.); 003856@hnucm.edu.cn (Y.L.); 2 Key Laboratory for Quality Evaluation of Bulk Herbs of Hunan Province, Hunan University of Chinese Medicine, Changsha 410208, China

**Keywords:** phenolic acids, hepatoprotective effect, liver disease, mechanisms

## Abstract

(1) Background: This article reviews the biological characteristics of phenolic acid compounds, focusing on their mechanisms of action in various liver diseases. (2) Methods: The review adheres to the Preferred Reporting Items for Systematic Reviews and Meta-Analyses (PRISMA) guidelines. We utilized PubMed and Web of Science databases to search for relevant studies on the use of phenolic acids in liver diseases from 2015 to 2025. (3) Results: Phenolic acids can improve different types of liver diseases, including drug-induced liver injury (DILI), alcoholic liver disease (ALD), metabolic dysfunction-associated steatotic liver disease, liver fibrosis, and liver cancer. Their beneficial effects are attributed to mechanisms such as anti-inflammatory properties, antioxidant activity, regulation of lipid metabolism, inhibition of cell apoptosis, and modulation of gut microbiota. (4) Conclusion: Phenolic acids exhibit a good protective effect against various liver diseases and are associated with multiple signaling pathways. However, the primary target cells and specific molecular targets of phenolic acids remain unclear, necessitating further research to elucidate their protective mechanisms in liver diseases.

## 1. Introduction

The liver is the largest and most important organ in the human body, with basic functions such as metabolism, secretion, and detoxification. Liver diseases cause more than 2 million deaths annually, accounting for 4% [1] of all deaths worldwide and have become a major cause of death. With the improvement in living standards, the prevalence of metabolic liver diseases, including nonalcoholic fatty liver disease (NAFLD) and ALD, is constantly rising, ultimately leading to an increase in cases of end-stage liver disease (liver fibrosis and liver cancer) [2]. Currently, no study has specifically analyzed the real incidence of DILI worldwide, but in follow-up studies, the proportion of DILI patients caused by herbal medicines and dietary supplements has been increasing [3].

Common hepatoprotective drugs in clinical practice include silibinin, ursodeoxycholic acid (UDCA), glycyrrhetinic acid preparations, bicyclol, and others. Silymarin is a substance extracted from *Silybum marianum* and is a widely used hepatoprotective drug in clinical settings. It exerts a good protective effect on various diseases such as NAFLD, ALD, and hepatitis [4]. However, caution is still required not only for the use of silymarin but also for other hepatoprotective drugs like glycyrrhizic acid preparations and bicyclol. This is because there are still very few clinical studies on their appropriate dosages and actual efficacy in different types of diseases. UDCA is the only drug approved by the U.S. Food and Drug Administration (FDA) for the treatment of primary biliary cholangitis. It was originally identified as a naturally active product from the bile of polar bears, and its current acquisition mainly relies on chemical or biosynthetic approaches for preparation. Nevertheless, the preparation process of UDCA still poses a major challenge in terms of cost and environmental protection [5]. Despite significant progress made in the field of drug research and development, there is no fully effective drug that can enhance liver function, provide whole-organ protection, or promote hepatocyte regeneration. Therefore, research on safer, more effective, and more economical hepatoprotective drugs is of crucial importance.

Phenolic acids are a class of bioactive substances widely present in nature, belonging to aromatic secondary metabolites. According to the differences in carbon skeleton and the position and quantity of hydroxyl groups on the aromatic ring, they can be classified into hydroxybenzoic acids (protocatechuic acid, vanillic acid, gallic acid, 4-hydroxybenzoic acid) and hydroxycinnamic acids (chlorogenic acid, ferulic acid, caffeic acid, rosmarinic acid) [6]. And a large number of studies have reported the hepatoprotective effects of phenolic acids [7]. Compared with existing clinical hepatoprotective drugs, phenolic acids, as naturally derived compounds, have potential advantages such as easy accessibility, low cost, and multiple pharmacological activities. However, they also have current limitations including low bioavailability and a lack of clinical trials.

**Figure 1:**
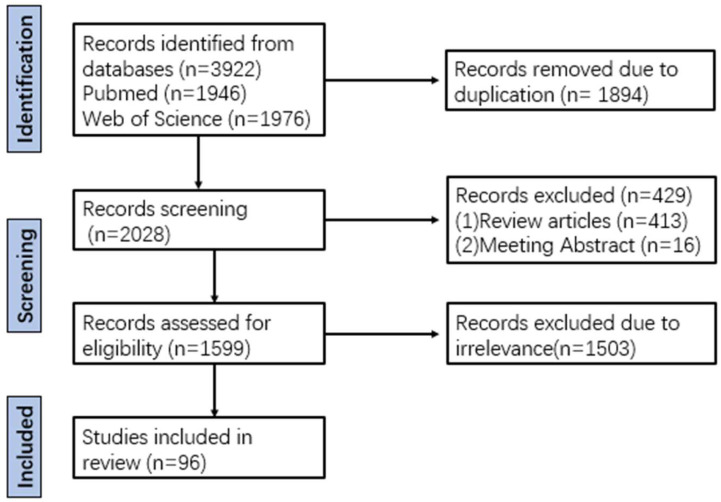
Flow diagram of literature search according to PRISMA guidelines.

However, most current studies focus on reports of individual phenolic acid monomers in single diseases, and there is a lack of systematic sorting and summary of the mechanisms underlying the hepatoprotective effects of phenolic acids, which limits their application. Therefore, this review followed the PRISMA guidelines. Several literature databases were searched, including PubMed and Web of Science, for papers published from 2015 to 2025. Keywords included “drug-induced liver injury”, “alcoholic liver disease”, “metabolic (dysfunction) associated fatty liver disease”, “liver fibrosis”, “liver cancer”, “phenolic acid” and “polyphenols”. Duplicate articles were excluded, and review articles and conference abstracts were excluded, along with unrelated studies, resulting in a final count of 96 eligible papers (Figure 1). Their pharmacological activities and related mechanisms were sorted out, aiming to provide a valuable theoretical basis for the research and development of phenolic acids in liver diseases and reduce the socioeconomic burden of liver diseases.

## 2. Biological Characteristics of Phenolic Acids

### 2.1. Chemical Structure of Phenolic Acids

Through literature review, it was found that there are 24 types of phenolic acid components with relevant hepatoprotective effects, mainly consisting of hydroxybenzoic acids and hydroxycinnamic acids. Among them, there are 13 types of hydroxybenzoic acids and 11 types of hydroxycinnamic acids.

**Figure 2:**
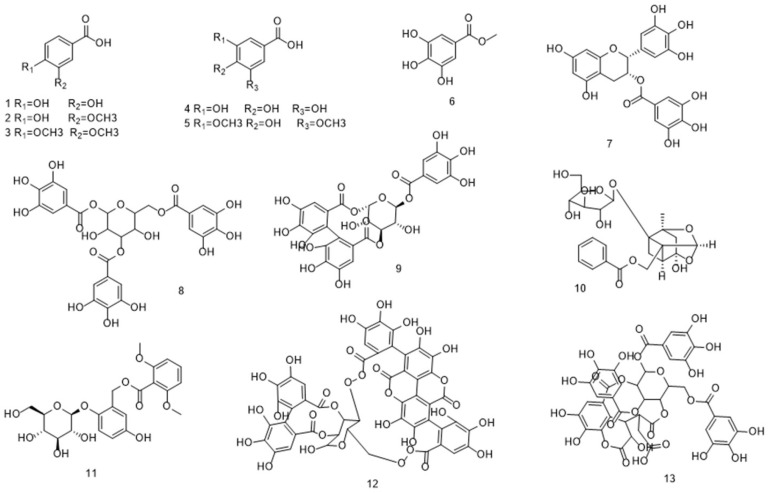
The structure of hydroxybenzoic acid 1. Protocatechuic acid 2. Vanillic acid 3. Veratric acid 4. Gallic acid 5. Syringic acid 6. Methyl gallate 7. EGCG 8. Tannic acid 9. Corilagin 10. Paeoniflorin 11. Curculigoside 12. punicalagin 13. Chebulinic acid.

Phenolic acids with a hydroxybenzoic acid skeleton are collectively referred to as hydroxybenzoic acids, which can be classified into simple hydroxybenzoic acids, condensed hydroxybenzoic acids, hydroxybenzoic acid esters, and hydroxybenzoic acid glycosides based on their structures. It has been reported that among these phenolic acids with hepatoprotective effects, simple hydroxybenzoic acids are the main type, including protocatechuic acid, vanillic acid, gallic acid, and veratric acid. Meanwhile, hydroxybenzoic acid esters, such as methyl gallate and epigallocatechin-3-gallate (EGCG), have been the subject of most studies. Their structures are shown in Figure 2.

**Figure 3:**
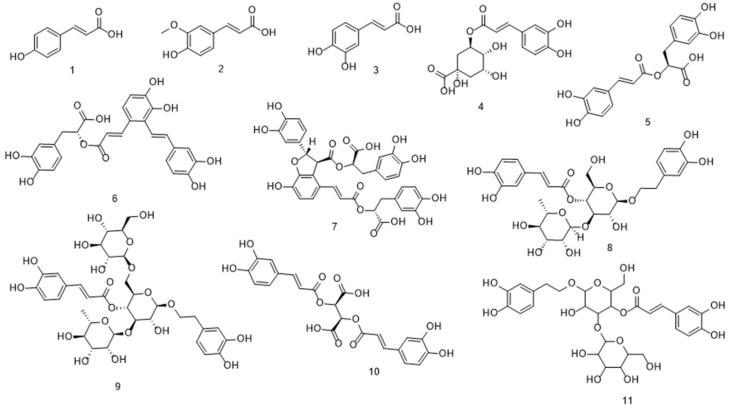
Structure of hydroxycinnamic acid 1. p-Coumaric acid 2. Ferulic Acid 3. Caffeic acid 4. Chlorogenic acid 5. Rosmarinic acid 6. Salvianolic Acid A 7. Salvianolic acid B 8. Acteoside 9. Echinacoside 10. Chicoric acid 11. Plantamajoside.

Hydroxycinnamic acids can be classified by structure into simple hydroxycinnamic acids, hydrogenated hydroxycinnamic acids, condensed hydroxycinnamic acids, hydroxycinnamic acid esters, hydroxycinnamic acid glycosides and hydroxycinnamate salts, among which simple hydroxycinnamic acids and condensed hydroxycinnamic acids are the most common types. It has been reported that among these phenolic acids with hepatoprotective effects, the most common simple hydroxycinnamic acids are p-coumaric acid, ferulic acid, and caffeic acid. Next are condensed hydroxycinnamic acids condensed with caffeic acid as the parent nucleus, including chlorogenic acid, rosmarinic acid, and chicoric acid; in addition, there are hydroxycinnamic acid esters and hydroxycinnamic acid glycosides such as salvianic acid A and salvianic acid B. Their structures are shown in Figure 3.

### 2.2. The Relationship Between Chemical Structure and Biological Activity

Studies on the relationship between compound structure and biological activity are of great significance for improving the targeting and pharmacokinetic properties of drugs, as well as for the research and development of new drugs. Phenolic acids are a class of aromatic compounds containing both phenolic hydroxyl groups and carboxylic acid groups. The number and position of hydroxyl groups are closely related to the activity of phenolic acids. A structure-activity relationship (SAR) study on phenolic acid compounds using the crocin bleaching assay revealed that the position of the carboxyl group relative to the hydroxyl substituent is crucial. Among hydroxybenzoic acids, gallic acid exhibits the strongest activity, which is 1.6 times and 3.4 times that of protocatechuic acid and syringic acid, respectively [8]. The SAR value of gallic acid, which has an additional hydroxyl group at the ortho position of the phenolic hydroxyl group, is more than twice that of protocatechuic acid (3.40 vs. 1.50). This indicates that the pyrogallol moiety in the molecular structure of phenolic compounds has better antioxidant activity than the catechol moiety [9,10]. This is because the ortho hydroxyl groups (electron donors) on the aromatic ring reduce the dissociation enthalpy of hydroxyl groups, thereby enhancing free radical scavenging activity [11].

In addition, special phenolic hydroxyl structures (such as ortho-dihydroxyl groups) and unsaturated bonds in side chains can also affect the activity of phenolic acids. The presence of free phenolic hydroxyl groups in caffeic acid reduces the dissociation enthalpy of hydroxyl groups, increases the hydrogen atom transfer rate of peroxyl radicals and the number of peroxyl radicals on the benzene ring, thereby enhancing the activity of phenolic acids. Furthermore, the presence of double bonds in the carbon chain increases the stability of phenolic hydroxyl radicals. These chemical factors related to caffeic acid are closely associated with its ability to scavenge free radicals and inhibit the production of reactive oxygen species (ROS) [12,13,14]. Meanwhile, some substituents can also enhance the activity of phenolic acids. Rosmarinic acid is an ester of caffeic acid and 3,4-dihydroxyphenyllactic acid. Previous studies have shown that rosmarinic acid has relatively weak antibacterial activity. However, among a series of successfully synthesized rosmarinic acid derivatives, RA-N8 (6-trifluoromethoxy-benzothiazol-2-yl) is the most effective antibacterial agent (against *Staphylococcus aureus* and *Escherichia coli*) [15]. In addition, rosmarinic acid can also form derivatives by combining with metal ions (sodium, silver), amine or imidazole, thereby exerting an anti-glioblastoma effect [16].

### 2.3. Pharmacokinetic Properties

Although phenolic acids are widely present in plant-based foods, their bioavailability is generally low. After ingestion, caffeic acid undergoes esterification; the esterified part of caffeic acid in the colon is cleaved by microbial esterases, and approximately 95% of it is then absorbed by the intestinal mucosa in free form. After absorption, enzymatic reactions (methylation, sulfation, and glucuronidation) occur to increase hydrophilicity, reduce toxic effects, and promote elimination [17]. Rosmarinic acid is easily absorbed from the gastrointestinal tract and through the skin and is distributed in various tissues. However, a small portion of rosmarinic acid is degraded into various components, such as glucuronic acid or sulfated conjugates of caffeic acid, ferulic acid, and trace amounts of coumaric acid, which are then rapidly excreted in urine [18]. The gastrointestinal tract has difficulty absorbing chlorogenic acid because neither the intestinal mucosa nor the intestinal flora possesses esterases that can cleave chlorogenic acid, due to the presence of the quinic acid moiety in the chlorogenic acid structure [19]. EGCG also has poor bioavailability. It is metabolized by the intestinal microbiota before absorption: it can be hydrolyzed into epigallocatechin (EGC) and gallic acid by rat intestinal bacteria and bacterial strains, followed by a series of transformations and degradation processes before being excreted from the body [20]. The main site of ferulic acid absorption in the human body is the colon; its absorption rate is fast, but the extent is very low. Intestinal microbial esterases hydrolyze esterified ferulic acid in food matrices to produce free ferulic acid, which is mainly excreted as feruloyl glucuronide [21]. In summary, the absorption and metabolism of phenolic acids are highly dependent on their structural characteristics, as well as the activity of intestinal esterases and intestinal flora. Caffeic acid and rosmarinic acid are well absorbed in the intestine. In contrast, chlorogenic acid, EGCG, and ferulic acid have low bioavailability. The lack of intestinal esterases and rapid pre-absorption metabolism by the intestinal flora reduces their absorption.

## 3. Molecular Mechanisms of Phenolic Acids in Modulating Liver Diseases

### 3.1. Antioxidant

Oxidative stress is caused by an imbalance between the production of ROS and antioxidant defense [22]. A substantial body of evidence suggests that oxidative stress plays a critical role in the pathophysiology of various liver diseases [23]. When exposed to stressors, the body’s antioxidant defense system is activated, in which nuclear factor erythroid 2-related factor 2 (Nrf2) acts as the primary regulator [24,25]. The dissociation of Kelch-like ECH-associated protein 1 (Keap1) from Nrf2 enables Nrf2 to translocate into the nucleus, where it binds to small Musculoaponeurotic fibrosarcoma oncogene homolog (MAF) proteins that associate with promoters containing Antioxidant response elements (ARE) [26,27]. This binding regulates the expression of a series of antioxidant factors, such as Heme oxygenase 1 (HO-1), NAD(P)H: Quinone oxidoreductase 1 (NQO1), Glutamate cysteine ligase catalytic (GCLC), and Glutamate cysteine ligase modifier (GCLM). Phenolic acids exert their antioxidant effects mainly by activating the Nrf2 pathway.

Research has shown that various phenolic acids can alleviate liver damage by activating Keap1/Nrf2 to exert antioxidant effects. For example, p-Coumaric acid, Caffeic acid, Rosmarinic acid, Veratric acid, and Chlorogenic acid can activate Nrf2 and upregulate the expression of downstream antioxidant proteins (HO-1, NQO1, GCLC, and GCLM), thereby playing a hepatoprotective role [28,29,30,31]. In the exploration of the antioxidant mechanism of phenolic acids, it has been found that phenolic acids can also regulate Nrf2 by acting on upstream pathways, including the AMP-activated protein kinase (AMPK)/Glycogen synthase kinase 3β (GSK3β) and Phosphoinositide 3-kinase (PI3K)/Protein kinase B (AKT) signaling pathways. Both chicoric acid and corilagin can increase the levels of Phospho-AMP-activated protein kinase α (P-AMPKα) and Phospho-Glycogen synthase kinase 3β (P-GSK3β), thereby activating Nrf2 and alleviating Lipopolysaccharides (LPS)/D-Galactosamine (GalN)-induced Acute liver failure (ALF) and Acetaminophen(APAP)-induced hepatotoxicity [32,33]. Paeoniflorin activates Nrf2 through the PI3K/AKT-dependent pathway, improving Alpha-Naphthylisothiocyanate (ANIT)-induced cholestatic liver injury in rats [34]. In addition, phenolic acids can exert antioxidant effects by targeting MicroRNA (miRNA). For instance, protocatechuic acid can reduce the formation of ROS through the MicroRNA-219a-5p (miR-219a-5p)/p66shc signaling pathway, thereby alleviating ALD [35]. Salvianolic acid A regulates the MicroRNA-485-3p (miR-485-3p)/Sirtuin 1 (SIRT1) pathway to mitigate APAP-induced oxidative stress [36].

Phenolic acids exhibit protective effects against liver injury induced by various drugs, particularly DILI caused by drugs such as APAP, Isoniazid, Rifampicin, Tetrachloromethane (CCl_4_), and Methotrexate (MTX). Impaired liver metabolic capacity leads to sustained oxidative stress in the liver; caffeic acid, gallic acid, and punicalagin can achieve hepatoprotective effects by exerting antioxidant activity [37,38,39,40]. DILI includes acute and chronic injury. For anti-tuberculosis drugs like isoniazid and rifampicin—which have a marked tendency to induce liver injury and require long-term administration—hepatoprotective drugs are generally co-administered in clinical practice to prevent liver injury. Currently, most animal experiments focus on preventive administration, so the therapeutic effect on unknown acute liver injury remains unclear. Drugs such as CCl_4_ and MTX are frequently used in animal experiments but lack sufficient clinical relevance.

**Figure 4:**
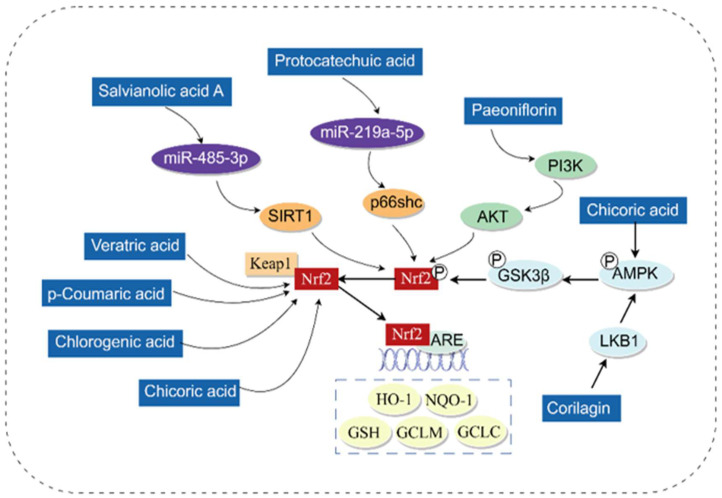
The molecular mechanism of phenolic acids exerting antioxidant activity via the Nrf2 pathway (By Figdraw). The dissociation of Keap1 from Nrf2 promotes the nuclear translocation of Nrf2, which then binds to ARE to enhance the expression of antioxidant factors. Multiple pathways, such as AMPK/GSK3β, PI3K/AKT, miR-219a-5p/p66shc, and miR-485-3p/SIRT1, can activate this process. Nrf2, Nuclear factor erythroid 2-related factor 2; Keap1, Kelch-1ike ECH-associated protein l; ARE, Antioxidant response elements; GCLC, Glutamate cysteine ligase catalytic; GCLM, Glutamate cysteine ligase modifier; NQO1, NAD (P) H: Quinone oxidoreductase 1; SOD, Superoxide dismutase; AKT, Protein kinase B; PI3K, Phosphoinositide 3-kinase; AMPK, AMP-activated protein kinase; GSK3β; Glycogen synthase kinase 3β; LKB1, Liver kinase B1; miR-485-3p, MicroRNA-485-3p; miR-219a-5p, MicroRNA-219a-5p.

The molecular mechanism by which phenolic acids exert antioxidant activity through the Nrf2 pathway is shown in Figure 4.

### 3.2. Anti-Inflammatory

When the liver is damaged by various etiologies (such as alcohol, hepatitis virus, lipid accumulation, cholestasis, etc.), the inflammatory response is a crucial link in the liver’s initiation of self-repair mechanisms. Nuclear factor kappa-light-chain-enhancer of activated B cells (NF-κB) is a key transcriptional regulator of the inflammatory response. Pathogen-associated molecular patterns (PAMPs) such as LPS, as well as Damage-associated molecular patterns (DAMPs) including High mobility group box 1 (HMGB1), can all activate NF-κB by acting on Toll-like receptors (TLRs), especially Toll-like receptor 4 (TLR4), leading to liver inflammation [41]. Studies have found that phenolic acids alleviate liver inflammation and mitigate liver diseases by inhibiting the NF-κB pathway.

(1) A variety of phenolic acids can reduce liver inflammation by inhibiting the TLR4/NF-κB pathway. For example, Chlorogenic acid and EGCG alleviate liver inflammation by directly inhibiting TLR4, thereby slowing the progression of nonalcoholic steatohepatitis (NASH) and liver injury caused by *Pseudomonas aeruginosa* infection [42,43]. Plantamajoside and chlorogenic acid inhibit the expression of Interleukin-1 Receptor-Associated Kinase 1 (IRAK1) and TNF Receptor-Associated Factor 6 (TRAF6), preventing NF-κB from translocating into the nucleus. This reduces the mRNA transcription of inflammatory factors and alleviates liver inflammation [44,45]. Protocatechuic acid and punicalagin can decrease the levels of pro-inflammatory cytokines such as Interleukin-6 (IL-6) and Tumor necrosis factor-α (TNF-α), thereby improving liver injury [40,46].

(2) In addition, as a DAMP, HMGB1 can be passively released from necrotic cells or actively secreted from immunocompetent cells during inflammation [47]. It interacts with TLR4 to further activate NF-κB, exacerbating the inflammatory response. Gallic acid, rosmarinic acid, paeoniflorin, and chlorogenic acid all exert anti-inflammatory effects by inhibiting the HMGB1/NF-κB/TLR4 pathway [38,48,49,50].

(3) On the other hand, the activation of NF-κB also induces the transcription of NOD-, LRR- and pyrin domain-containing protein 3 (NLRP3). This enables the NLRP3 inflammasome to recruit the adaptor protein Apoptosis-associated Speck-like protein containing a CARD (ASC) and Caspase 1, inducing the assembly of the NLRP3 inflammasome. Consequently, this activates Caspase 1 through autocatalysis and promotes the maturation and secretion of pro-inflammatory cytokines [51]. Both EGCG and chlorogenic acid can inhibit the activation of the NLRP3 inflammasome by downregulating the expression of NLRP3, ASC, and Interleukin-1β (IL-1β), thereby exerting hepatoprotective effects [31,52].

Meanwhile, phenolic acids can also inhibit Macrophage type 2 (M2) macrophage polarization, reduce the inflammatory infiltration of hepatic macrophages, and alleviate the inflammatory response. Combined treatment with metformin and p-coumaric acid, as well as combined administration of geniposide and chlorogenic acid, can improve liver inflammation by promoting the transition of macrophages from the Macrophage type 1 (M1) phenotype to the M2 phenotype, thereby inhibiting the inflammatory infiltration of hepatic macrophages [53,54]. Notably, opposite results have been observed in liver fibrosis models. Corilagin mediates the repolarization of M2 macrophage to M1 macrophage by inhibiting the expression of Indoleamine 2,3-Dioxygenase 1 (IDO1), thereby improving CCl_4_-induced liver fibrosis [55]. This is because chronic diseases such as chronic viral hepatitis, ALD, and NASH often lead to an imbalance between M1 and M2 responses. Activated M2 macrophages promote the activation of hepatic stellate cells (HSCs), resulting in liver fibrosis. This reflects the complexity of the inflammatory microenvironment during liver injury and repair: in the early stage of injury, the focus may be on inhibiting the pro-inflammatory M1 type, while in the repair stage, promoting the anti-inflammatory M2 type is more critical. Most existing studies fail to clearly define the temporal and spatial context of their interventions. Future research should not be limited to achieving phenotypic changes but must deeply reveal the specific immune microenvironment and molecules that drive such changes.

**Figure 5:**
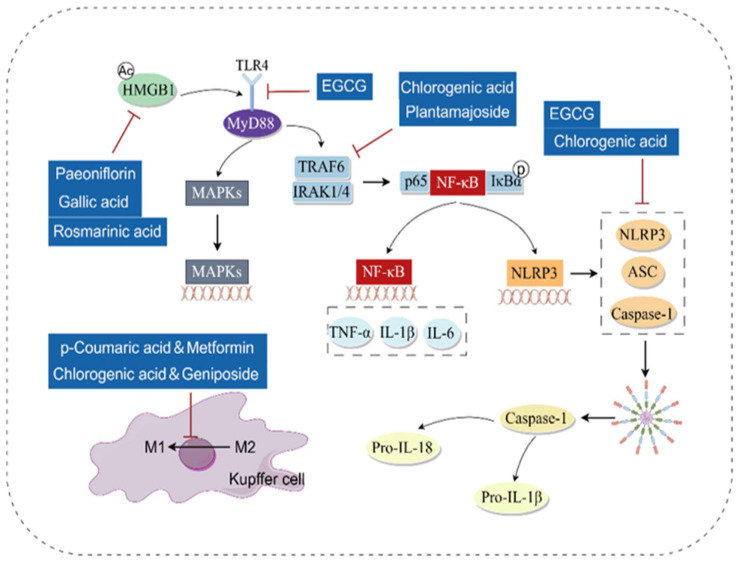
Molecular mechanism of anti-inflammatory action of phenolic acids (By Figdraw). After being activated by HMGB1, TLR4 binds to MyD88, thereby activating downstream IRAK1 and TRAF6. This process promotes the phosphorylation of NF-κB and facilitates its nuclear translocation. On one hand, this leads to the release of inflammatory factors; on the other hand, the activation of NF-κB promotes the transcription of NLRP3, which in turn facilitates the formation of the NLRP3 inflammasome. Phenolic acids primarily exert anti-inflammatory effects by inhibiting this process, and they can also act by promoting the conversion of M1 macrophage to M2 macrophage. TLR4, Toll-like receptor 4; MyD88, Myeloid Differentiation Primary Response 88; IRAK1/4, Interleukin-1 Receptor-Associated Kinase 1/4; TRAF6, TNF Receptor-Associated Factor 6; NF-κB, Nuclear factor kappa-light-chain-enhancer of activated B cells; IκB-α, Inhibitor of κB-alpha; ERK, Extracellular signal-regulated kinase; MAPK, Mitogen-activated protein kinase; TNF-α, Tumor necrosis factor-α; IL-6, Interleukin-6; IL-1β, Interleukin-1β; ASC, Apoptosis-associated Speck-like protein containing a CARD; NLRP3, NOD-, LRR- and pyrin domain-containing protein 3; Pro-IL-1β, Pro-form of Interleukin-1β; Pro-IL-18, Pro-form of Interleukin-18.

The molecular mechanism by which phenolic acids exert anti-inflammatory effects through the NF-κB pathway is shown in Figure 5.

### 3.3. Regulating Lipid Metabolism

An increase in fatty acids (FAs) delivered by adipose tissue, accompanied by an imbalance between lipid degradation and De novo lipid (DNL) synthesis, leads to excessive lipid accumulation in the liver and triggers the development of various liver diseases [56,57]. Phenolic acids can promote FAs β-oxidation and inhibit DNL by regulating pathways such as the (AMP)-activated protein kinase (AMPK)/Carnitine Palmitoyltransferase 1 (CPT1) pathway, AMPK/Sterol Regulatory Element-Binding Protein 1 (SREBP-1) pathway, and Peroxisome Proliferator-Activated Receptors (PPARs) pathway.

After FAs in the liver bind non-covalently to Fatty Acid Binding Protein-1 (FABP-1), CPT1 converts them into acylcarnitines, which are then shuttled into mitochondria. There, these acylcarnitines undergo β-oxidation to produce energy [58]. As a cellular “energy sensor”, the activation of AMPK phosphorylates and inhibits the activity of Acetyl CoA Carboxylase (ACC), leading to a decrease in malonyl-CoA levels. This, in turn, relieves the inhibition of CPT1 activity and promotes FAs entry into mitochondria for β-oxidation [59]. Combined treatment with metformin and p-coumaric acid, as well as rosmarinic acid, can promote FAs β-oxidation by activating AMPK-mediated CPT1 [53,60]. At the cellular level, studies have also shown that arctigenin chlorogenic acid from burdock roots enhances FAs β-oxidation through the AMPK/ACC/CPT1 pathway, thereby alleviating oleic acid-induced steatosis in Human Hepatoma G2 (HepG2) cells [61]. SIRT1 is a class III Nicotinamide Adenine Dinucleotide (NAD+)-dependent histone/protein deacetylase. A decrease in its activity may reduce the deacetylation of LKB1 and inhibit this kinase [62], which in turn regulates the activation of AMPK [63] and ultimately affects FAs metabolism. Salvianolic acid A upregulates the protein levels of phosphorylated AMPK and SIRT1 in a dose-dependent manner, improving hepatic lipotoxicity in mice with fatty liver [64]. Neochlorogenic acid alleviates lipid accumulation by downregulating MicroRNA-34a (miR-34a), thereby activating the SIRT1/AMPK pathway to improve oleic acid-induced intracellular lipid accumulation [65].

Phenolic acids can act on the AMPK/SREBP-1 pathway to inhibit lipogenesis and lipid accumulation. SREBP-1 is a key transcription factor for lipogenic genes [66]. AMPK phosphorylates SREBP-1 and inhibits its transcriptional activity, reducing the protein expression of ACC and Fatty Acid Synthase (FASN) [67,68], thereby suppressing DNL. Combined use of chicoric acid and fish oil can reduce palmitic acid-induced cellular lipid accumulation by regulating the AMPK-mediated SREBP-1/FASN pathway [69]. EGCG, chicoric acid, chlorogenic acid and combined use with metformin can all activate the p-AMPK/AMPK, downregulate the expression of Sterol Regulatory Element-Binding Protein 1c (SREBP1C) and its downstream lipogenesis-related targets (ACC and FASN), thereby alleviating NAFLD [70,71,72].

In addition, phenolic acids can act on the PPARs pathway to promote FAs β-oxidation. PPARs are transcription factors of the nuclear receptor family that regulate the gene transcription of key enzymes involved in FAs β-oxidation [73]. Among them, PPARα is mainly expressed in the liver. Studies have shown that chlorogenic acid can upregulate the hepatic expression of Adiponectin Receptor 2 (AdipoR2), increase the level of C1q/TNF-Related Protein 3 (CTRP3), and upregulate hepatic PPARα expression, thereby exerting hypoglycemic and lipid-regulating effects [74]. p-Coumaric acid activates PPARα to upregulate Hormone-Sensitive Lipase (HSL), Hepatic Triglyceride Lipase (HTGL), Monoacylglycerol Lipase (MGL), and Acyl-CoA Synthase Long-Chain 1 (ACSL1), thereby reducing serum and hepatic lipids and inhibiting the fusion and growth of lipid droplets [75]. Echinacoside can alleviate hepatic steatosis by upregulating PPARα [76]. Methyl ferulate can also promote the expression of PPARα and CPT1 by upregulating SIRT1, significantly inhibiting ethanol-induced hepatic steatosis [77]. ZFP30 is a member of the Krüppel-associated box zinc finger protein (KRAB-ZFP) family; recent studies have shown that chlorogenic acid inhibits the upregulation of ZFP30 in NAFLD and promotes the expression of PPARα, thereby enhancing FAs β-oxidation and alleviating hepatic steatosis [78]. Peroxisome Proliferator-Activated Receptor γ (PPARγ) is mainly expressed in adipose tissue. Peroxisome Proliferator-Activated Receptor Gamma Coactivator-1α (PGC-1α) is a transcriptional coactivator of PPARγ and is involved in the transcriptional regulation of PPARγ. Rosmarinic acid significantly alleviates NAFLD by repairing mitochondrial damage and regulating the PPARγ/PGC-1α signaling pathway [79].

Beyond the aforementioned common mechanisms, phenolic acids can also regulate FAs β-metabolism through pathways such as the Epidermal Growth Factor Receptor (EGFR)-AKT-mechanistic Target of Rapamycin (mTOR) pathway and Forkhead Box O1 (FoxO1) pathway. Salvianolic acid A inhibits the nuclear translocation of Carbohydrate Response Element-Binding Protein (ChREBP) by suppressing Thioredoxin-Interacting Protein (TXNIP), thereby improving high-fat diet (HFD)-induced hepatic steatosis [80]. Tannic acid and vitamin E-loaded-poly D, L-lactide-co-glycolic acid (PLGA) nanoparticles prevent hepatic steatosis and improve liver injury in a chronic alcoholic liver injury model by inhibiting the EGFR-AKT-mTOR pathway [81]. FoxO1 is a member of the FoxO family and plays an important role in hepatic steatosis [77]. Methyl ferulate downregulates the phosphorylated expression of p38 Mitogen-Activated Protein Kinase (p38 MAPK), c-Jun N-Terminal Kinase (JNK), Extracellular Signal-Regulated Kinase (ERK), and FoxO1 in L-02 cells, inhibiting ethanol-induced hepatic steatosis [77]. Rosmarinic acid exerts lipid-lowering effects by increasing the expression of ATP Binding Cassette G5 (ABCG5), ATP Binding Cassette G8 (ABCG8), and Cholesterol 7α-Hydroxylase A1 (CYP7A1) [60].

**Figure 6:**
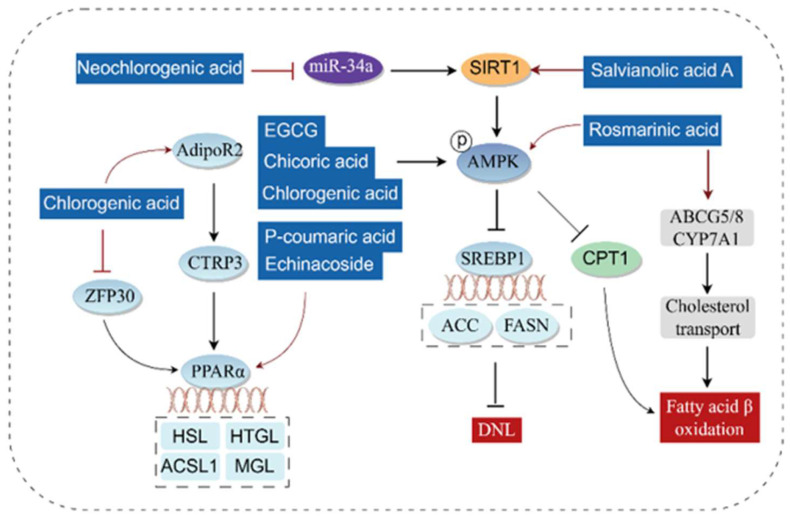
Molecular mechanisms of phenolic acids regulating lipid metabolism via the AMPK/ACC, AMPK/CPT1, and PPARs Pathways (By Figdraw). Phosphorylation of AMPK inhibits the transcriptional activity of SREBP1, thereby reducing the expression of ACC and FASN and ultimately suppressing DNL. Phosphorylation of AMPK promotes the expression of CPT1, which in turn enhances FAs β-oxidation; meanwhile, inhibition of miR-34a activates the SIRT1/AMPK pathway to regulate lipid metabolism. On the other hand, increased nuclear transcriptional activity of PPARα upregulates the transcription and expression of FA oxidases, thereby promoting fatty acid oxidation. The upregulation of CTRP3 and AdipoR2, as well as the downregulation of ZFP30, all promote the nuclear transcription of PPARα. FASN, Fatty acid synthase; CPT1, Carnitine palmitoyltransferase 1; SIRT1, Sirtuin 1; ACC, Acetyl CoA carboxylase; SREBP-1, Sterol Regulatory Element-Binding Protein 1; AMPK, AMP-activated protein kinase; CTRP3, C1q/TNF-related protein 3; AdipoR2, adiponectin receptor 2; ZFP30, Zinc finger protein 30; PPARα, Peroxisome proliferator-activated receptorα; HSL, Hormone-Sensitive Lipase; HTGL, Hepatic Triglyceride Lipase; MGL, Monoacylglycerol Lipase; ACSL1, Acyl-CoA Synthase Long-chain 1; DNL, De novo lipogenesis.

The molecular mechanism by which phenolic acids regulate lipid metabolism is shown in Figure 6.

### 3.4. Regulating Autophagy

Autophagy is an evolutionarily conserved cellular degradation process [82] which plays a key role in maintaining cellular homeostasis by degrading protein aggregates, pathogens, lipids, and aged/damaged subcellular organelles (e.g., damaged mitochondria) [83]. Dysfunction of hepatic autophagy may lead to various liver diseases, including NAFLD, DILI, cholestasis, and hepatocellular carcinoma (HCC). Autophagy can be classified into microautophagy, macroautophagy, and chaperone-mediated autophagy. Phenolic acids primarily exert hepatoprotective effects by regulating the macroautophagy pathway. This process mainly involves the following key steps: (a) formation or nucleation of phagophores; (b) conjugation of Autophagy-related gene 5—Autophagy-related gene 12 (Atg5-Atg12), interaction with Autophagy-related gene 16-like (Atg16L), and polymerization of phagophores; (c) processing of Microtubule-associated protein 1A/1B-light chain 3 (LC3) and its insertion into the membrane of elongating phagophores; (d) fusion of autophagosomes with lysosomes, followed by proteolytic degradation of engulfed molecules via lysosomal proteases.

The hepatoprotective effect of phenolic acids via autophagy is mainly achieved by promoting LC3 processing and regulating autophagy-related proteins such as Beclin1, PTEN-induced kinase 1 (PINK1), and Parkin.

(1) Beclin1 is a key protein involved in autophagy. It participates in the vesicle nucleation process by forming the Beclin1-Vps34 complex with Vacuolar protein sorting 34 (Vps34), Vacuolar protein sorting 15 (Vps15), and Autophagy-related gene 14-like (ATG14L) [84]. Punicalagin increases hepatic Beclin1 expression and the number of hepatic autophagosomes, thereby inhibiting insulin resistance in HFD-fed mice [85].

(2) After autophagy induction, LC3 is hydrolyzed to generate LC3I. Subsequently, LC3I is activated by Autophagy-related gene 7 (Atg7) and transferred to Autophagy-related gene 3 (Atg3). Finally, LC3I conjugates with Phosphatidylethanolamine (PE) via carboxyglycine to form processed LC3II [86]. Punicalagin, EGCG, and corilagin promote the processing of LC3II, upregulate autophagy, and alleviate diabetic liver injury, as well as liver injury mediated by APAP and HFD [85,87,88].

(3) PINK1 can translocate to the outer mitochondrial membrane to recruit Parkin [89], and is then ubiquitinated by Parkin. This ubiquitinated complex is subsequently recognized by Sequestosome 1 (p62/sequestosome 1) [90,91], which bridges mitochondria targeted for autophagy to LC3 on the autophagosome surface, thereby driving the elongation of phagophore membranes. In addition to PINK1-mediated autophagy, BCL2/adenovirus E1B interacting protein 3-like (BNIP3L) can also mediate autophagosome formation. Punicalagin increases the protein expression of hepatic P62 and BNIP3, enhances the number of hepatic autophagosomes, and inhibits insulin resistance and diabetic liver injury [92]. Ferulic acid activates the autophagic pathway by increasing the levels of PINK1, Parkin, and P62 [93].

(4) Beyond directly regulating autophagy-related proteins, in-depth studies have shown that certain phenolic acid components can also regulate autophagy by acting on signaling pathways such as AKT/Forkhead box O3 (FoxO3a), ROS/MAPK, and Janus kinase 2 (JAK2)/Signal transducer and activator of transcription 3 (STAT3). Punicalagin inhibits the phosphorylation of AKT/FoxO3a, upregulates the expression of the autophagy-related protein LC3B, and downregulates p62 expression, thereby protecting against liver injury induced by type 2 diabetes [94] and CCl_4_ [39]. EGCG promotes autophagy via the ROS/MAPK pathway [95]. Rosmarinic acid enhances autophagy by acting on the JAK2/STAT3 pathway [96].

In summary, most existing studies determine the role of phenolic acids in regulating autophagy by detecting autophagy-related proteins and generally lack comprehensive monitoring of the autophagic process. Moreover, excessive autophagy can lead to cellular self-digestion, making it difficult to determine whether the observed autophagy activation exerts a protective effect. Additionally, vanillic acid alleviates liver fibrosis by inhibiting autophagy in HSCs via the Macrophage Migration Inhibitory Factor (MIF)/Cluster of differentiation 74 (CD74) signaling pathway [97]. This study provides a crucial reverse perspective: it relieves fibrosis by inhibiting autophagy in HSCs, which contrasts sharply with the pro-autophagic effect of most phenolic acids in hepatocytes. Future research should further explore how phenolic acids coordinate the autophagic status of different cell types to achieve overall therapeutic effects in complex systems with multiple liver cell types coexisting, and clarify the precise connection between their upstream signals (e.g., Akt/FoxO3a, ROS/MAPK) and core autophagic mechanisms.

**Figure 7:**
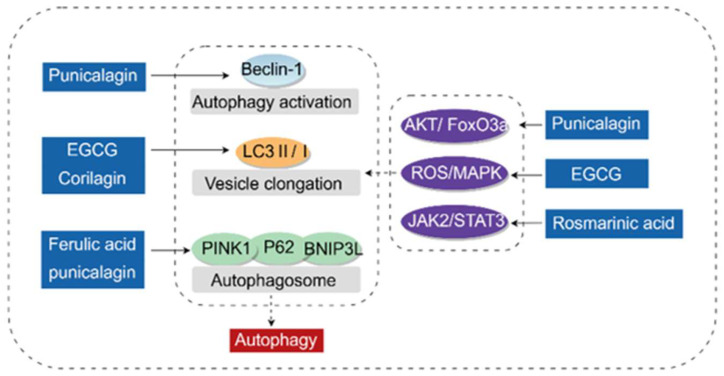
Molecular mechanism of phenolic acids regulating autophagy (By Figdraw). Beclin-1 is a key protein for activating autophagy; LC3I is processed into LC3 II, which then conjugates with PE (Phosphatidylethanolamine) to form LC3 II-PE. Subsequently, LC3 II-PE participates in the formation of phagophores together with BNIP3L (BCL2/adenovirus E1B interacting protein 3-like) and PINK1 (PTEN-induced kinase 1). LC3 II/I, Microtubule-associated protein 1A/1B-light chain 3 II/I; PINK1, PTEN-induced kinase 1; P62/SQSTM1, Sequestosome 1; BNIP3L, BCL2/adenovirus E1B interacting protein 3-like; AKT, AKT serine/threonine kinase; FoxO3a, Forkhead box O3a; JAK2, Janus Kinase 2; STAT3, Signal Transducer and Activator of Transcription 3; ROS, Reactive oxygen species; MAPK, Mitogen-activated protein kinase.

The molecular mechanism by which phenolic acids regulate autophagy is shown in Figure 7.

### 3.5. Regulating Apoptosis

Apoptosis is the primary mode of cell death in various liver diseases, characterized by an increased protein degradation rate [98,99] and elevated caspase activity [100]. Caspase activation can be triggered by two well-characterized apoptotic pathways: the mitochondria-mediated (intrinsic) pathway and the cell surface death receptor (extrinsic) pathway [101]. Phenolic acids mainly exert anti-apoptotic effects by activating the intrinsic pathway.

The B-cell lymphoma 2 (BCL-2) family consists of proteins with pro-apoptotic and anti-apoptotic properties, and plays a crucial role in the mitochondria-mediated intrinsic pathway [102]. The pro-apoptotic protein BAX induces mitochondrial outer membrane permeabilization, leading to the release of cytochrome c from the mitochondrial intermembrane space into the cytosol [103]. Cytochrome c then binds to Apoptotic protease-activating factor 1 (Apaf-1) to form a multimeric Apaf-1/cytochrome c complex. This complex recruits Procaspase-9, resulting in the formation of the apoptosome [104]. Subsequently, Procaspase-9 is activated via proteolysis [103], leading to its dissociation from the complex, and then activates Caspases 3, 6, and 7 [104]. The extrinsic apoptotic pathway relies on the activation of cell surface death receptors, which induce receptor trimerization and the recruitment of specific intracellular receptor-associated proteins such as Procaspase-8. Procaspase-8 is immediately cleaved into Caspase-8, which activates downstream effector caspases, ultimately leading to cell apoptosis [105].

Studies have shown that phenolic acids can exert hepatoprotective effects in liver injury models induced by drugs, toxic substances, etc., by regulating apoptosis-related proteins. Particularly in DILI, for example, APAP-induced DILI is commonly used to study hepatocellular necrosis. APAP is mainly metabolized into glucuronide and sulfate conjugates; a small portion is converted by the CYP enzyme family into the toxic metabolite N-acetyl-p-benzoquinone imine (NAPQI). NAPQI causes glutathione (GSH) depletion and ROS release, ultimately leading to cell death. (1) Ferulic acid, paeoniflorin, and EGCG can all improve DILI by regulating the expression of apoptosis-related proteins, including BAX, BCL-2, Caspase 3, and Caspase 9 [106,107,108]. Punicalagin can alleviate liver injury caused by acrylamide (ACR), arsenic trioxide (ATO), and methotrexate (MTX) by inhibiting apoptosis [40,109,110]. The combined use of chlorogenic acid, rutin, and quercetin can also reduce triptolide (TP)-induced liver injury by inhibiting apoptosis [111]. (2) In addition to apoptosis induced by chemical substances, veratric acid, paeoniflorin, and chlorogenic acid can also inhibit liver ischemia/reperfusion (I/R) injury, ANIT-induced cholestasis, and liver fibrosis by downregulating BAX, upregulating BCL-2, and reducing the expression of cleaved Caspase-3 and cleaved caspase-9 [50,112,113].

In summary, the mechanisms by which phenolic acids regulate apoptosis all boil down to the modulation of “BAX/BCL-2” and “Caspase-3/9”. Although this demonstrates the hepatoprotective effects of phenolic acids, no studies have clarified how different phenolic acids specifically interfere with the initial upstream signals of this pathway. Secondly, the vast majority of evidence from existing studies comes from DILI models (e.g., APAP-induced), which are characterized by acute cellular necrosis and apoptosis. However, in chronic liver diseases, cell death is a continuous process, and there is currently a lack of robust evidence to confirm whether phenolic acids can still exert protective effects through a single anti-apoptotic mechanism in these more complex pathological environments that are closer to clinical reality. Additionally, there is a dynamic transition between apoptosis and necrosis. In models such as APAP-induced hepatotoxicity, inhibiting the apoptotic pathway may also shift the mode of cell death to more inflammatory necrosis, inadvertently exacerbating liver injury. Existing studies generally fail to simultaneously monitor multiple modes of cell death, which makes the evaluation of the overall biological consequences of the anti-apoptotic effects of phenolic acids incomplete.

**Figure 8:**
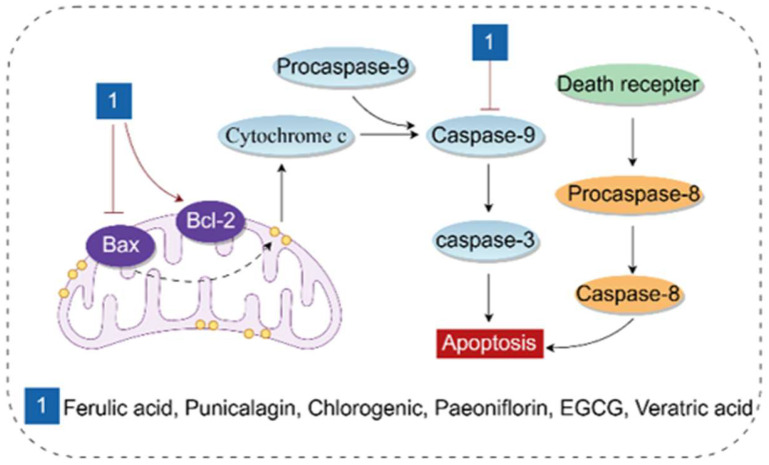
Molecular mechanism of phenolic acids inhibiting apoptosis (By Figdraw). The pro-apoptotic protein BAX induces the release of cytochrome c from the outer mitochondrial membrane, which subsequently recruits procaspase-9. The hydrolysis of procaspase-9 leads to the dissociation of caspase-9 and the activation of caspase-3, ultimately resulting in cell apoptosis. By contrast, the extrinsic apoptotic pathway relies on the activation of cell surface death receptors, which promotes the activation of caspase-8 to induce cell apoptosis. BCL-2, B-cell lymphoma 2; BAX, BCL2-associated X.

The molecular mechanism of phenolic acids inhibiting apoptosis is shown in Figure 8.

### 3.6. Anti-Fibrosis

Liver fibrosis is caused by excessive production of the extracellular matrix (ECM) and abnormal deposition of fibrous connective tissue in the liver. The massive production and activation of myofibroblasts (MFBs)—which secrete components including collagen, α-smooth muscle actin (α-SMA), and tissue inhibitor of metalloproteinases 1 (TIMP1) [114,115]-are critical to the progression of liver fibrosis, and MFBs mainly originate from the activation of HSCs. Studies have shown that phenolic acids can exert anti-fibrotic effects by regulating the transforming growth factor β (TGF-β)/Smad pathway, HMGB1/TLR4/NF-κB pathway, and targeting the platelet-derived growth factor receptor (PDGFR).

Numerous studies have reported that phenolic acids inhibit liver fibrosis by regulating the TGF-β/Smad pathway. TGF-β is a key cytokine driving liver fibrosis [116]. When injury occurs, the secretion of TGF-β increases in the fibrotic microenvironment and promotes Smad2/3 activation through TGF-β receptor 1/2 (TGFBR1/2) [117]. Subsequently, phosphorylated Smad2/3 forms an oligomeric complex with Smad4, translocates into the nucleus, and regulates the transcription of target genes, thereby mediating fibrosis [118]. Smad7 is a negative feedback regulator of TGF-β signaling [119], and increasing Smad7 expression can delay liver fibrosis [120,121,122]. Ferulic acid can attenuate TGF-β1-induced HSCs activation by inhibiting collagen I, Smad2/3 phosphorylation, and Smad4 signal transduction [123]. Chlorogenic acid inhibits the expression of miR-21, α-SMA, and TGF-β, while increasing the protein expression of Smad7 and matrix metalloproteinase-9 (MMP-9), thereby alleviating liver fibrosis [124]. Echinacoside exerts anti-fibrotic effects by inhibiting the TGF-β1/Smad signaling axis mediated by activin A receptor, type II A (ACVR2A) [125]. In vitro experiments have also confirmed that protocatechuic acid significantly reduces the cell viability of HSCs and downregulates the protein expression of TGF-β and phosphorylated Smad2 [126].

In recent years, PDGFR has become a promising target for anti-fibrotic drugs [127,128]. Activation of the platelet-derived growth factor-BB homodimer (PDGF-BB)/PDGFRβ pathway exhibits a significant pro-fibrotic response [129]. Studies have shown that salvianolic acid B may inhibit HSCs activation by targeting PDGFRβ and significantly reduce the levels of α-SMA and collagen I [130]. Chlorogenic acid prevents liver fibrosis by inhibiting the PDGF-induced pro-fibrotic effect via the NAD(P)H oxidase (NOX)/ROS/MAPK pathway [50].

**Figure 9:**
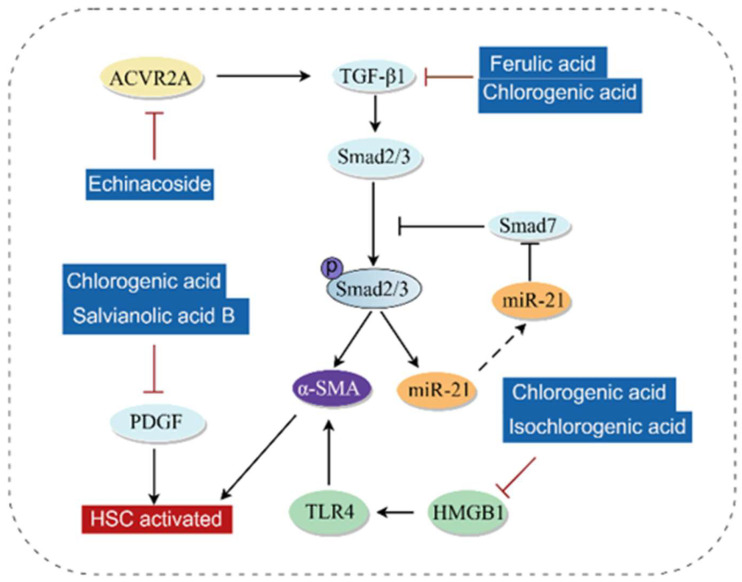
Molecular mechanism of phenolic acids against liver fibrosis (By Figdraw). When injury occurs, the secretion of TGF-β1 in the fibrotic microenvironment increases, activating Smad2/3 and promoting its phosphorylation. After translocating into the nucleus, phosphorylated Smad2/3 promotes the expression of miR-21 and α-SMA. Once miR-21 translocates out of the nucleus, it inhibits the expression of Smad7. The HMGB1/TLR4 pathway can upregulate the expression of α-SMA, thereby promoting HSC activation. Activation of PDGF can also promote HSC activation. TGF-β1, Transforming growth factor β1; PDGF, Platelet-derived growth factor; HMGB1, High mobility group box 1; α-SMA, α-smooth muscle actin; TLR4, Toll-like receptor 4; miR-21, MicroRNA-21; ACVR2A, Activin A receptor, type II A; HSCs, Hepatic stellate cells.

Phenolic acids can also inhibit the development of liver fibrosis by acting on the HMGB1/TLR4/NF-κB pathway. In addition to excessive ECM accumulation, persistent chronic inflammation also drives fibrosis [131,132]. Existing evidence indicates that inhibiting HMGB1 significantly reduces the inflammatory response and liver fibrosis [133]. Therefore, targeting the signaling pathway between HMGB1 and its receptors may provide a new approach to alleviate liver fibrosis. Isochlorogenic acid and chlorogenic acid can inhibit the protein expression of HMGB1 and TLR4, suppress the overexpression of α-SMA in HSCs, and prevent HSCs activation, thereby alleviating liver fibrosis [134,135]. However, these studies still lack mechanistic depth and cannot confirm that phenolic acids alleviate liver fibrosis by inhibiting HMGB1. In the author’s view, phenolic acids only inhibit the expression of fibrosis-related proteins while reducing inflammation. Since inflammation is one of the drivers of fibrosis, targeting key targets such as TGF-β is obviously a more promising therapeutic strategy. The molecular mechanism of phenolic acid compounds against liver fibrosis is shown in Figure 9.

### 3.7. Regulating Gut Microbiota and Gut–Liver Axis

The intestinal barrier protects the body from potentially toxic metabolites, bacteria, and their antigens. Therefore, the intestinal barrier and the immune control of the symbiotic microbiota are of crucial importance [136]. Based on the gut–liver axis, the intestinal microbiota regulates pro-inflammatory changes in the liver and intestines, thereby influencing the development of hepatitis, liver fibrosis, cirrhosis, and hepatocellular carcinogenesis [137].

Many phenolic acids contribute to this multifaceted and dynamic host microbe symbiosis. EGCG [138], punicalagin [92], Chicoric Acid [29], TLP-H [139] (gallic acid, corilagin and chebulagic acid) and Chlorogenic [140] can reduce the ratio of *Firmicutes/Bacteroidetes* and decrease the abundance of co-bacteria and anaerobic bacteria. Caffeic acid also helps balance intestinal microbiota dysbiosis associated with LPS [141]. TLP-H, EGCG, and Chlorogenic acid can also significantly alleviate intestinal dysbiosis and liver inflammation by inhibiting the activation of the TLR4/MyD88/NF-κB signaling pathway, thereby alleviating liver injury [139,140,142]. Chlorogenic acid can increase the expression of occludin and zonula occludens-1 (ZO-1) in intestinal tissues to regulate the intestinal microbiota; in addition, it can reduce the level of LPS in the portal vein and increase the level of glucagon-like peptide-1 (GLP-1) to exert anti-inflammatory effects [30].

**Figure 10:**
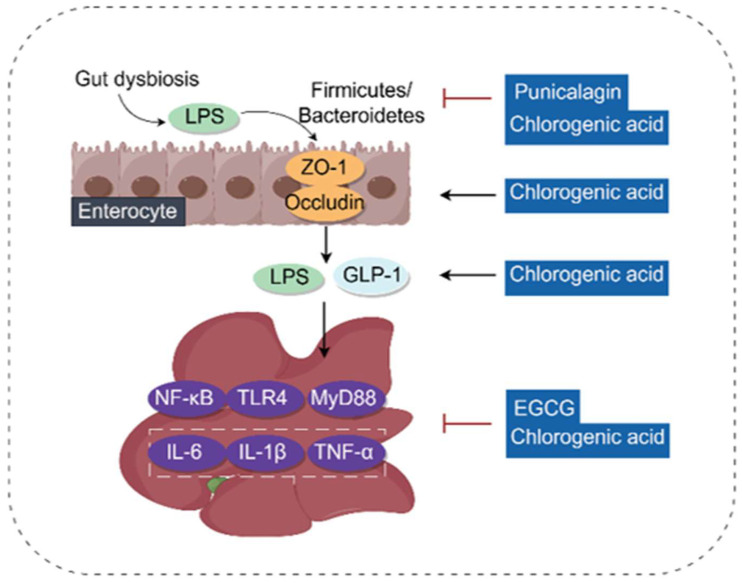
Molecular mechanism of phenolic acids regulating gut microbiota (By Figdraw). Impairment of the intestinal barrier allows PAMPs such as LPS to enter the liver via the bloodstream. Once in the liver, these PAMPs activate the NF-κB pathway, ultimately leading to the release of inflammatory factors. On the other hand, the release of LPS also induces intestinal microbiota dysbiosis, which in turn downregulates the protein levels of zonula occludens-1 (ZO-1), occludin, and glucagon-like peptide-1 (GLP-1) in the intestine. TLR4, Toll-like receptor 4; MyD88, Myeloid Differentiation Primary Response 88; NF-κB, Nuclear factor kappa-light-chain-enhancer of activated B cells; LPS, Lipopolysaccharides; ZO-1, Zonula occludens-1; GLP-1, Glucagon-like peptide-1; TLR4, Toll-like receptor 4; MyD88, Myeloid Differentiation Primary Response 88; NF-κB, Nuclear factor kappa-light-chain-enhancer of activated B cells; TNF-α, Tumor necrosis factor-α; IL-6, Interleukin-6; IL-1β, Interleukin-1β.

In summary, we observe that phenolic acid treatment is accompanied by improved microbiota composition, intestinal barrier repair, and alleviated liver injury. However, very few studies have directly confirmed that specific microbiota changes are a necessary cause for the hepatoprotective effects of phenolic acids through experiments such as fecal microbiota transplantation or gene-knockout animal models. Furthermore, an unavoidable translational medicine challenge lies in the contradiction between the bioavailability of phenolic acids and their local intestinal concentration. Many phenolic acids are poorly absorbed in the upper intestine, which, conversely, allows them to reach the large intestine at higher concentrations to interact with the microbiota. Future research must clarify whether their hepatoprotective effects rely on systemic pharmacological effects after absorption, or on influencing the liver through signal transmission (e.g., via GLP-1) or metabolite absorption after exerting local effects in the intestine. This is crucial for deciding whether to develop derivatives that enhance systemic exposure or to design formulations targeting colonic delivery. The molecular mechanism of phenolic acids regulating gut microbiota is shown in Figure 10.

### 3.8. Other Mechanisms

In addition to the mechanisms mentioned above, phenolic acids can also exert hepatoprotective effects through biological processes such as epigenetic regulation, endoplasmic reticulum (ER) stress modulation, ferroptosis inhibition, suppression of HCC cell proliferation, promotion of liver repair and regeneration, and pyroptosis regulation.

## 4. Clinical Translation Prospects of Phenolic Acids

Phenolic acids are abundant in plant-derived foods and exhibit high safety. For example, chlorogenic acid is one of the most accessible phenolic acid compounds; it exists naturally and in high concentrations in raw coffee extracts and tea [143], and possesses pharmacological activities such as antibacterial, antiviral, and antioxidant effects. Grains like rice, wheat, and oats are the main sources of ferulic acid, which is also present in various fruits and vegetables. In Japan, ferulic acid is used as a food additive, antioxidant, and food preservative, with a long history of safe consumption [144]. Various berries, such as blueberries, are rich in gallic acid, caffeic acid, protocatechuic acid, and more [145]. However, this does not mean that phenolic acids are absolutely safe when used as therapeutic drugs. Studies have shown that rosmarinic acid is non-toxic to humans, even when patients take a dose of 500 mg/day [146]; yet, the safety of its long-term application remains unknown.

Low oral bioavailability is a common challenge faced by most phenolic acids. Due to their large molecular weight and high polarity, some phenolic acids are difficult to pass through the intestinal mucosa via passive diffusion, resulting in poor intestinal absorption. Those phenolic acids that are absorbed undergo Phase II conjugation metabolism in the intestines and liver, converting into metabolites that are excreted from the body. This leads to extremely low concentrations of the parent drug entering the systemic circulation. To address this bottleneck, numerous studies have shown that advanced dosage forms such as nanotechnology (e.g., liposomes, polymeric nanoparticles), phospholipid complexes, and emulsion-based delivery systems can be used to improve the bioavailability of phenolic acids. Chitosan is a naturally occurring biopolymer; the binding of natural bioactive molecules (e.g., polyphenols) to chitosan can enhance bioactivity and water solubility [147]. Research has demonstrated that the caffeic acid-chitosan conjugate exerts a significant hepatoprotective effect against ethanol-induced hepatotoxicity in mice [148]. Additionally, with the development of nanotechnology, the pharmaceutical and biotechnology industries have gradually explored new directions to substantially improve the efficacy of antioxidants. Gold nanoparticles (AuNPs) have attracted great interest due to their biocompatibility, colloidal stability, water solubility, and catalytic activity. SAuPTB (AuNP-tannic acid hybrid nanozymes) exhibit strong antioxidant activity [149].

Significant pharmacokinetic differences in phenolic acids exist between humans and animals, this means that dose conversion between animals and humans cannot simply rely on conversion formulas. Studies have shown that the maximum therapeutic dose of chlorogenic acid in a rat model of liver fibrosis is 60 mg/kg, which converts to an approximate adult equivalent dose of 580 mg/day—far higher than the daily dietary intake of humans. In a randomized, double-blind clinical trial (*n* = 101), researchers administered chlorogenic acid (200 mg/day) to patients with non-alcoholic fatty liver disease (NAFLD) for 6 months. They found that compared with the placebo group, there was no significant reduction in liver biochemical indicators; however, this dose was completely safe without any side effects [150]. Therefore, one of the top priorities of future clinical research is to determine the true pharmacokinetic characteristics of phenolic acids in the human body. It may also be necessary to improve their bioavailability through novel delivery systems, thereby making the effective doses based on animal models truly clinically relevant.

Although numerous animal and cell experiments have confirmed the hepatoprotective effects of phenolic acids, it is undeniable that design flaws in preclinical studies exist. Widely used cell lines (e.g., HepG2, HEK293) undergo genetic and phenotypic drift during long-term passage, and their biological characteristics differ significantly from primary human cells. Animal models typically “induce” diseases in a short period through gene knockout or strong chemical/physical stimulation. For instance, CCl_4_ injection or bile duct ligation can induce liver fibrosis within a few weeks. This differs from the multifactorial, long-term, and chronic disease progression process in humans and cannot fundamentally replicate the actual disease development. In animal studies, drugs are usually administered either simultaneously with model induction (preventive use) or immediately after model establishment (therapeutic use). In contrast, clinical patients typically begin treatment only after the disease has been established, or even after fibrosis or carcinogenesis has occurred. Organoid [151] and Patient-Derived Tumor Xenograft (PDX) [152] models can enhance the clinical relevance of research.

## 5. Discussion

Liver diseases pose a significant challenge to global public health, characterized by complex pathogenic mechanisms and limited therapeutic options. In recent years, phenolic acid compounds have emerged as a research hotspot in liver disease studies due to their diverse pharmacological activities. In clinical settings, there have been epidemiological studies on the association between phenolic acid intake and metabolic diseases such as diabetes [153], hypertension [154], and metabolic syndrome [155]. There are few clinical studies on phenolic acids for liver diseases beyond their use as dietary supplements. In a cross-sectional study, the correlation between phenolic acid intake and the risk of NAFLD was analyzed among 9894 enrolled adults. The results showed that participants with higher dietary phenolic acid intake had a lower risk of developing NAFLD [156]. Some Chinese patent medicines rich in salvianolic acids, such as Danshen Tablets and Salvia Miltiorrhiza Ligustrazine Injection, have been clinically used for liver diseases including hepatitis and fatty liver. Although clinical applications of individual phenolic acid monomers are limited, these findings highlight the potential importance of phenolic acid-rich dietary patterns as a component of strategies to reduce liver disease risk.

This article reviews the pharmacological reports on the hepatoprotective effects of phenolic acids in vivo and in vitro. Among them, hydroxycinnamic acids have been the most extensively studied, including caffeic acid, ferulic acid, rosmarinic acid, and chlorogenic acid. For hydroxybenzoic acids, EGCG has garnered the widest research attention. These compounds are not only effective in various liver injury models but, more importantly, can simultaneously regulate multiple core nodes of disease networks, making them recommended as priority candidates for in-depth development as potential drugs.

Prioritize the Development of the Most Promising Lead Compounds. Our analysis reveals that chlorogenic acid and EGCG have the most extensive research and relatively abundant evidence in the field of hepatoprotection. Existing literature has comprehensively summarized their dietary sources, bioavailability, pharmacological effects, and potential targets. However, clinical trial data on these compounds remain scarce. In a randomized controlled trial, NAFLD patients were administered Altilix^®^ (Bionap, Belpasso, Catania, Italy; a formulation containing chlorogenic acid and its derivatives, as well as luteolin and its derivatives) for 6 months. Compared with the placebo group, significant reductions in liver function indicators were observed [157]. Meanwhile, active exploration of the synergistic therapeutic potential of standardized extracts rich in multiple phenolic acids (e.g., Altilix^®^) is warranted, as such multi-component formulations may offer advantages over single-component agents.

Focus on the Most Core and Validated Mechanisms. At present, the primary target cells and specific key molecular targets of phenolic acids for hepatoprotection remain unclear. Taking chlorogenic acid, the most extensively studied phenolic acid, as an example, it exhibits various pharmacological effects such as anti-inflammation, antioxidant activity, regulation of lipid metabolism, and anti-liver fibrosis. However, compared with other phenolic acids that also possess these pharmacological effects, this does not reflect the specificity of the regulatory targets of phenolic acids, nor how different phenolic acids precisely and preferentially regulate specific links among these processes. In recent years, research on the gut-liver axis has advanced significantly. Phenolic acids can exert broad-spectrum therapeutic effects by improving intestinal microbiota dysbiosis, enhancing intestinal barrier function, and regulating bile acid metabolism. Furthermore, based on individual differences in intestinal microbiota, the development of combined phenolic acid-prebiotic [158] therapies may be feasible in the future. These findings suggest that attention should be paid to crosstalk between multiple organs. Integrating multi-omics technologies (e.g., proteomics and transcriptomics) with clinical cases will help identify new key targets, facilitate in-depth mechanistic studies, and achieve precise regulation.

Overcoming Core Bottlenecks in Clinical Translation. Optimize delivery systems to address bioavailability challenges. Priority must be given to developing liver-targeted delivery systems based on nanotechnology (e.g., liposomes, polymeric nanoparticles) to increase their accumulation concentration in the liver. Conduct standardized preclinical and clinical studies. Before initiating human trials, systematic toxicological and pharmacokinetic evaluations complying with Good Laboratory Practice (GLP) standards must be completed. Subsequently, rigorous Phase I and Phase II clinical trials should be designed to first verify the safety and preliminary efficacy of phenolic acids in high-risk populations with NAFLD or DILI. Advance precision medicine and rational combination therapy. Meanwhile, explore combination therapy strategies of phenolic acids with existing standard drugs (e.g., metformin, obeticholic acid) to exert synergistic effects, reduce the dosage of each drug, and minimize toxic side effects. The possible mechanisms by which phenolic acids modulate liver diseases are shown in Table 1.

Therefore, we believe that future translational research and clinical trials can prioritize focusing on widely studied phenolic acids such as chlorogenic acid and EGCG, taking them as “lead compounds” for in-depth development and application exploration. At the same time, on the basis of promoting research on phenolic acid combination therapy and the application of dietary supplements, attention should also be paid to obtaining nutrients, antioxidants, bioactive compounds, or phytochemicals from a balanced diet, so as to achieve optimal nutrition, health, and well-being.

## Figures and Tables

**Table 1:** Molecular regulatory mechanism of phenolic acids in liver disease.

No	Component	Dose	Model	Mechanism	Pharmacological Activity	Reference
1	P-coumaric acid	10–100 µM	ALD	↑Nrf2, ↓MAPKs	Inhibit cell apoptosis, anti-inflammatory, antioxidant	[28]
2	Chicoric acid	15, 30 mg/kg	HFD	↑AMPK, ↑Nrf2, ↓NFκB	Anti-inflammatory, antioxidant,	[29]
3	chicoric acid	50 mg/kg	ALF	↑AMPK, ↑Nrf2, ↓NF-κB	Anti-inflammatory, antioxidant	[32]
4	Protocatechuic acid	10, 20 mg/kg 10 μM	ALD	↑miR-219a-5p, ↓p66shc, ↓ROS	Antioxidant	[35]
5	Salvianolic acid A	15, 30 mg/kg	APAP	↓miR-485-3p, ↑SIRT1	Anti-inflammatory, antioxidant	[36]
6	Caffeic acid	10, 30 mg/kg	APAP	↓Keap1, ↑Nrf2, ↑HO-1, ↑NQO1	Anti-inflammatory, antioxidant	[37]
7	Gallic acid	50, 100, 150 mg/kg	Isoniazid and rifampicin	↑Nrf2, ↓NF-κB	Antioxidant	[38]
8	Pomegranate extract	20, 40, 80 mg/kg	CCl_4_	↓AKT/FoxO3a, ↑Nrf2, ↑Beclin-1, ↓LC3-I/II	Promote autophagy, antioxidant	[39]
9	Pomegranate extract	25, 50 mg/kg	MTX	↑BCL-2, ↑Nrf2	Anti-inflammatory, antioxidant	[40]
10	Epigallocatechin gallate 3-gallate	20, 40, 80 mg/kg	Acute pneumonia	↓TLR4-myD88-NF-κB, ↑PXR, ↑AhR, ↑CAR	Anti-inflammatory, antioxidant	[43]
11	Plantago asiatica saponins	50, 100 mg/kg	CLP	↓TRAF6, ↓NF-κB	Inhibit cell apoptosis, Anti-inflammatory	[44]
12	Protocatechuic acid	15, 30 mg/kg	IR/D	↓NF-κB, ↓IL-1β, ↑Wnt1, ↑β-catenin, ↑GLUT4	Antioxidant, anti-inflammatory	[46]
13	Epigallocatechin gallate 3-gallate	0.32% EGCG (*w*/*v*)	Perfluorodecanoic acid	↓NLRP3	Anti-inflammatory	[52]
14	P-coumaric acid	200 mg/kg	NAFLD	↓NF-κB, ↑BAT, ↑sWAT	Inhibit cell apoptosis, anti-inflammatory, promote thermogenesis,	[53]
15	Corilagin	20 mg/kg 2.5, 5, 10, 20, 40 mM	NAFLD	↑LC3A/B II, ↓p62	Promote autophagy, antioxidant	[55]
16	Rosmarinic acid	50, 100 mg/kg	HFD	↑p-AMPK, ↑CPT1A ↑ABCG5, ↑ABCG8, ↑CYP7A1,	Inhibit lipid accumulation	[60]
17	Salvianolic acid A	20, 40 mg/kg	NAFLD	↑SIRT1, ↑AMPK, ↑LC3-II	Inhibit cell apoptosis, promote autophagy	[64]
18	Chicoric acid	100, 200 mM	Palmitate	↑PPARa/UCP2, ↓SREBP-1/FAS	Regulating lipid metabolism	[69]
19	Epigallocatechin gallate 3-gallate	100 mg/kg	NAFLD	↓FGF21, ↑FGFR/AMPK, ↑Nrf2	Antioxidant	[70]
20	P-coumaric acid	100 mg/kg 10 µg/mL	Hyperlipidemia	↑PPARα/γ, ↑AMPK, ↑MSS4	Regulating fat metabolism	[75]
21	Purple cone chrysanthemum glycoside	100 mg/kg		↑PPAR-α, ↓SREBP-1c/ FASN	Antioxidant, inhibits hepatic steatosis	[76]
22	Rosmarinic acid	100, 200 mg/kg 20, 40, 80 μmol/L	NAFLD	↓YAP1, ↓TAZ, ↑PPARγ, ↑PGC-1α	Antioxidant	[79]
23	Pomegranate extract	20 mg/kg	Diabetes	↑Bnip3, ↑Pink1, ↑Parkin	Promote mitochondrial autophagy, antioxidant,	[85]
24	Epigallocatechin gallate 3-gallate	0.6% EGCG 511 mg/kg	APAP	↑LC3B II/I, ↓BAX/BCL-2	Promote cell autophagy, inhibit cell apoptosis	[87]
25	Pomegranate extract	20 mg/kg	HFD	↓IKKβ/NF-κB	Regulating gut microbiota homeostasis, promoting liver autophagy, anti-inflammatory	[92]
26	Ferulic acid	0, 40, 60, 80, 100, 120, 140, 160 μg/mL		↓Caspase-3, ↑Beclin-1, ↓LC3-I/II	Promote cell apoptosis, autophagy	[93]
27	Pomegranate extract	20 mg/b.w	HFD	AKT/FoxO3a		[94]
28	Epigallocatechin gallate 3-gallate	50 mg/kg 50 μM	NAFLD	↓ROS, ↓p-JNK, ↓p-p38, ↓BAX/BCL-2, ↓Bad/Bcl-xl, ↑Beclin-1, ↑LC3	Inhibit cell apoptosis, promote autophagy	[95]
29	Rosmarinic acid	40, 80 mg/kg 5, 10, 20 μM	Toosendanin	↑JAK2/STAT3/CTSC	Promote autophagy	[96]
30	Vanillic acid	5, 20 mg/kg 5, 20 μM	Liver fibrosis	↓MIF, ↓CD74, ↓LC3B, ↓α-SMA	Inhibition of autophagy	[97]
31	Ferulic acid	25, 50, 100 mg/kg	APAP	↑HNF4a, ↑Foxa2, ↑ALB, ↑BAX/BCL2, ↑Caspase3, ↑p-AMPK	Antioxidant, anti-apoptotic, promoting autophagy	[106]
32	Epigallocatechin gallate 3-gallate	0.5, 1, 2 mg/kg 1, 2, 5, 10 μg/mL	APAP	↓NOS2, ↓MPO, ↓JNK, ↓BAX, ↑BCL-2	Anti-inflammatory, antioxidant, inhibition of cell apoptosis	[108]
33	Pomegranate extract	10, 20, 40 mg/kg	ACR	↑GSH, ↓MDA, ↓BAX/BCI-2, ↓Caspase3	Antioxidant, anti-apoptotic	[109]
34	Tannins	20, 40 mg/kg	ATO	↑Keap1-Nrf2/ARE, ↑BCL-2, ↓BAX, ↓Caspase-3	Anti-inflammatory, antioxidant, inhibition of cell apoptosis	[110]
35	Resveratrol acid	40, 80 mg/kg	I/R	↑Nrf2, ↑HO-1, ↑NQO-1	Antioxidant, anti-apoptotic	[112]
36	Chlorogenic acid	15, 30, 60 mg/kg 20, 40, 80 mg/mL	Liver fibrosis	↓TGF-β1/Smad7	Anti-inflammatory,	[124]
37	Purple cone chrysanthemum glycoside	10, 20 mg /kg	Liver fibrosis	↓TGF- β1/Smad	Anti-inflammatory, anti-liver fibrosis	[125]
38	Protocatechuic acid	75, 150 mg/kg 1 mM, 3 mM	TAA	TGF-β, ↓p-Smad2, ↓p-ERK, ↓c-Jun	Inhibiting cell apoptosis	[126]
39	Salvianolic acid B	25, 50 mg/kg	CCl_4_	↓PDGFRβ	Anti-inflammatory	[130]
40	Epigallocatechin gallate 3-gallate	50 mg/kg	MCD	↓ALT, ↓AST	Improve intestinal microbiota imbalance	[138]
41	Caffeic acid	10, 30, 50 mg /kg	CLP	↓NET, ↓5- LOX/LTB4	Anti-inflammatory, antioxidant, inhibition of cell apoptosis	[159]
42	Caffeic acid	10, 20 mg/kg	1,3-dichloro-2-propanol	↑Nrf2, ↓NF-kB	Anti-inflammatory, antioxidant	[160]
43	Gallic acid	50, 100 mg/kg	Diclofenac	↓IL-1β	Antioxidant	[161]
44	Gallic acid	5, 20 mg /kg	CLP	C/EBP β-Dependent MAPK	Anti-inflammatory	[162]
45	Gallic acid	100 mg/kg	TAA	↓Fas, ↓Caspase-3, ↓NF-κB	Antioxidant, anti-inflammatory, inhibition of cell apoptosis	[163]
46	Protocatechuic acid	10, 20 mg/kg	CCl_4_	↓NF-κB, ↓COX-2	Anti-inflammatory, antioxidant	[164]
47	Protocatechuic acid	100, 150 mg/kg	TAA	↓MDA, ↓NOx, ↑GSH, ↑SOD, ↓TNF-α, ↓IL-6, ↓IL-17, ↓IL-23, ↓Caspase3, ↓p53, ↓mTOR	Anti-inflammatory, antioxidant, inhibition of cell apoptosis, inhibition of autophagy	[165]
48	Protocatechuic acid	100, 150 mg/kg	Cisplatin	↓Caspase3, ↓annexin-V, ↓BAX, ↓iNOS, ↓IL-6, ↓TNF-α	Anti-inflammatory, antioxidant, inhibition of cell apoptosis	[166]
49	Protocatechuic acid	0.025%, 0.1% (*w*/*w*) PCA high-fat feed	HFD	↑*Fgf1*, ↑*Igfbp2*, ↑*Irs1*, ↑*Irs*	Improve insulin resistance, anti-inflammatory	[167]
50	Mulberry white skin (original catechins)	165 mg/kg 80 μM	Diabetes	↑PPARγ, ↑PI3K/Akt, ↓GSK3β, ↓FoxO1	Improve insulin resistance,	[168]
51	Vanillic acid	100 mg/kg	CCl_4_	↓ALT, ↓AST, ↑GSH, ↑SOD	Antioxidant	[169]
52	Vanillic acid	0.75, 75 mg/ kg	Liver cancer	↑GSTA-5, ↑Nrf2, Cyclin D1, ↑Caspase-3, ↓BAX, ↑BCL-2	Anti-proliferation, anti-apoptosis	[170]
53	Syringic acid	40, 80 mg/kg	SV	↓TNF-α, ↓C-JUN, ↓NF-κB, ↓COX-2, ↓IL-6	Anti-inflammatory, antioxidant	[171]
54	Epigallocatechin gallate 3-gallate	50 mg/kg	NIAAA	↓TLR2, ↑TLR 3, ↑IL-10, ↓NF-κB,	Anti-inflammatory, inhibits cell apoptosis	[172]
55	Epigallocatechin gallate 3-gallate	300 mg/kg	CCl_4_	↓TNF-α, ↓NF-κB, ↓IL-1β, ↓TGFβ, ↓p-ERK, ↓p-Smad1/2	Antioxidant, anti-inflammatory, anti-liver fibrosis	[173]
56	Epigallocatechin gallate 3-gallate	10, 20, 40, 80, 160 μg/mL		↑AKT, ↑JNK, ↑p53, ↑BAX, ↓BCL-2	Promote cell apoptosis	[174]
57	Epigallocatechin gallate 3-gallate	15 mg/kg	Cisplatin	↓p53, ↓BCL-2/BAX, ↓Caspase3	Promote cell apoptosis	[175]
58	Epigallocatechin gallate 3-gallate	0.3% (3 g/100 g)	NAFLD	↓LPS-TLR4-NFκB	Anti-inflammatory, improves microbiota imbalance, alleviates insulin resistance	[176]
59	Epigallocatechin gallate 3-gallate	50 mg/kg EGCG	FeSO4	↑Nrf2, ↑GPX4, ↑FTH/L	Antioxidant, regulating iron metabolism	[177]
60	Epigallocatechin gallate 3-gallate			↓PVT1, ↓TLR4, ↑miR-16-5p	Anti-inflammatory	[178]
61	Epigallocatechin gallate 3-gallate	0.4% EGCG (*w*/*w*)	NAFLD	↓ALT, ↓AST, ↓TNF-α, ↓COX-2, ↓MMP-3	Anti-inflammatory	[179]
62	Epigallocatechin gallate 3-gallate	0%, 0.1%, 0.5% EGCG	HCC	↓miR483-3p	Inhibit the metastasis of HCC	[180]
63	Epigallocatechin gallate 3-gallate	25 mg/kg 20 μmol/L	Liver cirrhosis and liver cancer	↓Hoshida, ↓α-SMA	Inhibition of HCC tumor formation	[181]
64	Epigallocatechin gallate methyl ester	25 mg/kg 25, 50, 75, 100, 125, 150 µg/mL	DEN	↑p21waf1/Cip1, ↓CDC25A	Inhibition of proliferation of human liver cancer cells	[182]
65	Methyl gallate	10, 20, 40 μg/mL		↓Caspase3, ↑BCI-2, ↓BAX, ↑Beclin-1, ↓LC3-I/II	Inducing cell apoptosis, promoting autophagy	[183]
66	Corilagin	5, 10 mg/kg	NAFLD	↓TC, ↓TG	Improve liver lipid accumulation	[184]
67	Corilagin	12.5, 25 mg/kg 1, 5, 10, 20 mg/mL	ConA	↓MAPK, ↓NF-kB, ↓IRF5	Anti-inflammatory, antioxidant	[185]
68	Mugaxanthin, gallic acid (crude leaf suspension and boiled leaf extract)	500 mg/kg	Liver fibrosis	↓TGF-β/SMAD	Anti-fibrosis	[186]
69	Chebullinic acid	37.5, 75, 150 mg/kg 6.5, 13, 26 µM	CCl_4_	↑MAPK/Nrf2, ↑LDH, ↑HO-1, ↑NQO1, ↓MDA, ↑SOD	Antioxidant	[187]
70	Pomegranate extract	100, 150, 200 mg/kg 5, 10, 20 mM	HFD	↑PI3K/AKT 28, ↓HMGB-1/TLR4/NF-κB	Reduce gluconeogenesis	[188]
71	Pomegranate extract	0.2% (*w*/*v*)	Fructose		Inhibited lipid deposition	[189]
72	Pomegranate extract	20 mg/kg	HFD	↑FoxO1, ↓TXNIP, ↓NLRP3, ↓Caspase1, ↓IL-1β, ↓GSDMD	Inhibit pyroptosis and promote autophagy	[190]
73	Curcumin saponins	20 mg/kg	I/R	↑Nrf-2/HO-1	Antioxidant, anti-inflammatory, apoptotic	[191]
74	Ferulic acid	50 mg/kg	Cesium-137	↓JAK/ STAT, ↑Nrf2, ↑GPX4, ↑SLC7A11	Anti-inflammatory, inhibits iron death, antioxidant	[192]
75	Ferulic acid	10, 30, 100 mg/kg	SA	↑PPAR-γ, ↑GLUT2	Anti-inflammatory, antioxidant	[193]
76	Salvianolic acid A	10, 20 mg/kg	IR	↓TLR4, ↑Caspase3	Antioxidant, anti-inflammatory, inhibition of cell apoptosis	[194]
77	P-coumaric acid	50, 100 mg/kg	Fipronil	↓ROS, ↓TNF-α, ↓IL-1β	Antioxidant, anti-inflammatory,	[195]
78	For coumaric acid and ferulic acid	30 mg/kg/day	HFD	↓HDAC1/PPARG	Inhibit lipid uptake	[196]
79	P-coumaric acid	100 mg/kg	LPS/D-GalN	↓TNF—α, ↓IL-6, ↓IL-1 β, ↓MDA, ↑GSH	Regulating lipid metabolism, antioxidant, anti-inflammatory	[197]
80	Arctiin	25, 50, 100 mg/kg	HIRI	↓HMGB1-TLR3/4-IRF1		[198]
81	Rosmarinic acid	20, 40, 80 mg/kg,	APAP	↑Nrf2, ↓NEK7-NLRP3	Anti-inflammatory, antioxidant	[199]
82	Rosmarinic acid			↑p-AMPK, ↓SREBP-1c	Anti-inflammatory	[200]
83	Rosmarinic acid	0.25, 1, 5 μM	H_2_O_2_	↑MAPK, ↑Nrf2	Antioxidant	[201]
84	Rosmarinic acid	20, 50, 100 μmol/L	SMMC-7721	↓PI3K/AKT/mTOR	Inhibit the proliferation and invasion of liver cancer cells	[202]
85	Rosmarinic acid	10, 20, 40 mg/kg	APAP	↓RACK1/TNF-a	Antioxidant	[203]
86	Rosmarinic acid	30, 90, 270 mg/kg	OVA	↓TNF-α, ↓IL-4, ↓IL-6, ↓mMCP-1, ↓iNOS	Antioxidant, anti-inflammatory	[204]
87	Rosmarinic acid	10, 30 mg/kg 20, 40 µM	NASH	↑p-AMPK, ↓SREBP-1c, ↓FAS	Antioxidant, anti-inflammatory	[205]
88	Paeoniflorin	25, 50, 100 mg/kg	Diabetes	↓TXNIP/NLRP3	Anti inflammatory	[206]
89	Paeoniflorin	20 mg/kg	HCD	↓FAS, ↓SREBP-1c, ↑p-AMPK, ↓p-MYPT-1	Antioxidant, anti-inflammatory	[207]
90	Paeoniflorin	50, 100, 200 mg/kg	APAP	↓CYP2E1/JNK	Antioxidant, anti-inflammatory	[208]
91	Paeoniflorin	100 mg/kg	Liver fibrosis	↑HO-1	Antioxidant, anti-inflammatory	[209]
92	Paeoniflorin	50, 100 mg/kg	Liver fibrosis	↑PPARγ	Inhibit HSC activation	[210]
93	Paeoniflorin	25, 100 mg/kg	Liver cancer	↑SOCS3↓STAT3/PD-L1	Inhibit the growth of liver cancer cells	[211]
94	Paeoniflorin	75 mg/kg	ZnO NPs	↓SIRT1, ↑mTOR, ↓TFEB, ↓NLRP3	Anti-inflammatory, improves intestinal microbiota and metabolic disorders	[212]
95	Paeoniflorin	15, 30, 60 mg/kg	LPS	↑SIRT1, ↑FOXO1a, ↑SOD2, ↓NLRP3	Anti-inflammatory, antioxidant	[213]
96	Purple cone chrysanthemum glycoside			↑miR-503-3p, ↓TGF-β1/Smad, ↑BAX/BCL-2	Inhibit the proliferation, invasion, migration of liver cancer cells	[214]

## Data Availability

No new data were created or analyzed in this study. Data sharing is not applicable to this article.

## Abbreviations

The following abbreviations are used in this manuscript:

**ABCG5**, ATP binding box G5; **ABCG8**, ATP binding box G8; **ACC**, Acetyl CoA carboxylase; **ACSL1**, Acyl-CoA Synthase Long-chain 1; **ACR**, Acrylamide; **ACVR2A**, Activin A receptor, type II A; **ATO**, Arsenic trioxide; **AdipoR2**, Adiponectin receptor 2; **Atg3**, Autophagy-related gene 3; **Atg7**, Autophagy-related gene 7; **AKT,** Protein kinase B; **ALF**, Acute liver failure; **AMPK**, AMP-activated protein kinase; **ANIT**, Alpha-Naphthylisothiocyanate; **APAP**, Acetaminophen; **ARE**, Antioxidant response elements; **ALD**, Alcoholic liver disease; **Atg5-Atg12**, Autophagy-related gene 5—Autophagy-related gene 12; **Atg16L**, Autophagy-related gene 16-like; **ATG14L**, Autophagy-related gene 14-like; **BAX**, BCL2-Associated X; **BCL-2**, B-cell lymphoma 2; **Bip**, Binding immunoglobulin protein; **BNIP3L**, BCL2/adenovirus E1B interacting protein 3-like; **CD74**, Cluster of differentiation 74; **CCl_4_**, Tetrachloromethane; **ChREBP**, Carbohydrate response element-binding protein; **CHOP**, C/EBP-homologous protein; **CTRP3**, C1q/TNF-related protein 3; **CPT1**, Carnitine Palmitoyltransferase 1; **CTSC**, Cathepsin C; **CYP7A1**, Cholesterol 7 α hydroxylase A1; **CLP**, Cecal ligation and puncture; **Con-A**, Concanavalin A; **DEN**, Diethylnitrosamine; **DAMPs**, Damage associated molecular patterns; **DILI**, Drug-induced liver injury; **DNL**, De novo lipogenesis; **ECM**, Extracellular matrix; **EGCG**, Epigallocatechin gallate; **EGC**, Epigallocatechin; **EGR1**,Early growth response 1; **ER**, Endoplasmic reticulum; **ERK**, Extracellular signal-regulated kinase; **EGFR**, Epidermal growth factor receptor; **FA**, Fatty acid; **FABP-1**, Fatty acid binding protein-1; **FASN**, Fatty acid synthase; **FDA,** Food and Drug Administration; **FPN**, Ferro-portin; ***Ftl***, *Ferritin light chain Gene*; **FoxO1**, Forkhead box O1; **FoxO3a**, Forkhead box O3; **GLP**, Good Laboratory Practice; **GLP-1**, Glucagon-like peptide-1; **GalN**, D-Galactosamine; **GCLC**, Glutamate cysteine ligase catalytic; **GCLM**, Glutamate cysteine ligase modifier; **GSH**, Glutathione synthase; **GSK3β**, Glycogen synthase kinase 3β; **HCC**, Hepatocellular carcinoma; **HMGB1**, High mobility group box 1; **HepG2**, Human hepatoma G2 cells; ***Hamp1***, *Hepcidin antimicrobial peptide Gene 1*; **HFD**, high-fat diet; **HFCD**, High-fat and high carbohydrate diet; **HCD**, High cholesterol diet.; **HIRI,** Hepatic ischemia–reperfusion injury; **HSC**, Hepatic stellate cell; **HSL**, Hormone-Sensitive Lipase; **HTGL**, Hepatic Triglyceride Lipase; **IDO1**, Indoleamine 2,3-Dioxygenase 1; **IR/D**, Insulin Resistance/Diabetes; **IL-6**, Interleukin-6; **IL-1β**, Interleukin-1β; **IRAK1**, Interleukin-1 Receptor-Associated Kinase 1; **I/R**, Ischemia/Reperfusion; **JAK2**, Janus kinase 2; **JNK**, c-Jun N-terminal kinase; **Keap1**, Kelch-1ike ECH-associated protein l; **KRAB-ZFP**, Krüppel-associated box zinc finger protein; **LPS**, Lipopolysaccharides; **LKB1**, Liver kinase B1; **LC3**, Microtubule-associated protein 1A/1B-light chain 3; **M2**, Macrophage type 2; **M1**, Macrophage type 1; **MAPK**, Mitogen-activated protein kinase; **MAFLD**, Metabolic dysfunction-associated fatty liver disease; **mTOR**, mechanistic target of rapamycin; **MGL**, Monoacylglycerol Lipase; **MAF**, Musculoaponeurotic fibrosarcoma oncogene homolog; **MFBs**, Myofibroblasts; **miRNA**, MicroRNA; **miR-485-3p**, MicroRNA-485-3p; **miR483-3p**, MicroRNA483-3p; **miR-17**, MicroRNA-17; **miR-503-3p**, MicroRNA-503-3p; **miR-219a-5p**, MicroRNA-219a-5p; **miR-34a**, MicroRNA-34a; **miR-21**, MicroRNA-21; **MTX**, Methotrexate; **MMP-9**, Matrix metalloproteinase-9; **MyD88**, Myeloid Differentiation Primary Response 88; **MCD**, Methionine and Choline Deficiency; **NAFLD**, Non-alcoholic fatty liver disease; **NASH**, Nonalcoholic Steatohepatitis; **NAD^+^**, Nicotinamide adenine dinucleotide; **NAPQI**, N-acetyl-p-Benzoquinone imine; **NF-κB**, Nuclear factor kappa-light-chain-enhancer of activated B cells; **NIAAA**, National Institute on Alcohol Abuse and Alcoholism; **NLRP3**, NOD-, LRR- and pyrin domain-containing protein 3; **NQO1**, NAD (P) H:Quinone oxidoreductase 1; **Nrf2**, Nuclear factor erythroid 2-related factor 2; **NOX**, NAD(P)H oxidase; **OVA**, Ovalbumin; **PE**, Phosphatidylethanolamine; **PINK1**, PTEN-induced kinase 1; **p62/SQSTM1**, Sequestosome 1; **PAMPs**, Pathogen associated molecular patterns; **P-AMPKα**, Phospho-AMP-activated protein kinase α; **PDGFR**, Platelet-derived growth factor receptor; **P-GSK3β**, Phospho-Glycogen synthase kinase 3β; **PGC-1α**, Peroxisome proliferator-activated receptor gamma coactivator-1α; **p38 MAPK**, p38 mitogen-activated protein kinase; **PI3K**, Phosphoinositide 3-kinase; **PDGF-BB**, Platelet-derived growth factor-B-B homodimer; **PDX**, Patient-derived tumor xenograft; **PPAR**, Peroxisome proliferator-activated receptor; **PLGA,** poly D, L-lactide-co-glycolic acid; **PPARα**, Peroxisome proliferator-activated receptor α; **PPARγ**, Peroxisome proliferator-activated receptor γ; **PRISMA**, Preferred Reporting Items for Systematic Reviews and Meta-Analyses; **RA-N8,** 6-trifluoromethoxybenzthiazol-2-yl; **ROS**, Reactive oxygen species; **SAR**, Structure-acitivity relationships; **STZ**, Streptozotocin; **SIRT1**, Sirtuin 1; **α-SMA**, α-smooth muscle actin; **SREBP-1**, Sterol regulatory element-binding protein 1; **STAT3**, Signal transducer and activator of transcription 3; **sXBP1**, Spliced X-box binding protein 1; **SV**, Sodium valproate; **SUMO1**, Small ubiquitin-like modifier 1; **TRAF6**, TNF Receptor-Associated Factor 6; **TREM2**, Triggering receptor expressed on myeloid cells-2; **TIMP1,** Tissue inhibitor of metalloproteinases 1; **TGF-β**, Transforming growth factor β; **TXNIP**, Thioredoxin-interacting protein; **TAA**, Thioacetamide; **TFR1**, Transferrin receptor 1; **TLRs**, Toll-like receptors; **TLR4**, Toll-like receptor 4; **TNF-α**, Tumor necrosis factor-α; **UDCA**, Ursodeoxycholic acid; **Vps34**, Vacuolar protein sorting 34; **Vps15**, Vacuolar protein sorting 15; **ZFP30**, Zinc finger protein 30; **ZO-1**, Zonula occludens-1.

## References

[1] Devarbhavi H., Asrani S.K., Arab J.P., Nartey Y.A., Pose E., Kamath P.S. (2023). Global burden of liver disease: 2023 update. J. Hepatol..

[2] Xiao J., Wang F., Wong N.K., He J., Zhang R., Sun R., Xu Y., Liu Y., Li W., Koike K. (2019). Global liver disease burdens and research trends: Analysis from a Chinese perspective. J. Hepatol..

[3] Andrade R.J., Chalasani N., Björnsson E.S., Suzuki A., Kullak-Ublick G.A., Watkins P.B., Devarbhavi H., Merz M., Lucena M.I., Kaplowitz N. (2019). Drug-induced liver injury. Nat. Rev. Dis. Primers.

[4] Camini F.C., Costa D.C. (2020). Silymarin: Not just another antioxidant. J. Basic Clin. Physiol. Pharmacol..

[5] Song P., Zhang X., Feng W., Xu W., Wu C., Xie S., Yu S., Fu R. (2023). Biological synthesis of ursodeoxycholic acid. Front. Microbiol..

[6] Sun Y., Ji X., Cui J., Mi Y., Zhang J., Guo Z. (2022). Synthesis, Characterization, and the Antioxidant Activity of Phenolic Acid Chitooligosaccharide Derivatives. Mar. Drugs.

[7] Simón J., Casado-Andrés M., Goikoetxea-Usandizaga N., Serrano-Maciá M., Martínez-Chantar M.L. (2020). Nutraceutical Properties of Polyphenols against Liver Diseases. Nutrients.

[8] Ordoudi S.A., Tsimidou M.Z. (2006). Crocin bleaching assay (CBA) in structure-radical scavenging activity studies of selected phenolic compounds. J. Agric. Food Chem..

[9] Kawabata J., Okamoto Y., Kodama A., Makimoto T., Kasai T. (2002). Oxidative dimers produced from protocatechuic and gallic esters in the DPPH radical scavenging reaction. J. Agric. Food Chem..

[10] Siquet C., Paiva-Martins F., Lima J.L., Reis S., Borges F. (2006). Antioxidant profile of dihydroxy- and trihydroxyphenolic acids—A structure-activity relationship study. Free Radic. Res..

[11] Wright J.S., Johnson E.R., DiLabio G.A. (2001). Predicting the activity of phenolic antioxidants: Theoretical method, analysis of substituent effects, and application to major families of antioxidants. J. Am. Chem. Soc..

[12] Sidoryk K., Jaromin A., Filipczak N., Cmoch P., Cybulski M. (2018). Synthesis and Antioxidant Activity of Caffeic Acid Derivatives. Molecules.

[13] Touaibia M., Jean-François J., Doiron J. (2011). Caffeic Acid, a versatile pharmacophore: An overview. Mini Rev. Med. Chem..

[14] Son S., Lewis B.A. (2002). Free radical scavenging and antioxidative activity of caffeic acid amide and ester analogues: Structure-activity relationship. J. Agric. Food Chem..

[15] Wang Y., Liang Z., Cao Y., Hung C.H., Du R., Leung A.S., So P.K., Chan P.H., Wong W.L., Leung Y.C. (2024). Discovery of a novel class of rosmarinic acid derivatives as antibacterial agents: Synthesis, structure-activity relationship and mechanism of action. Bioorg. Chem..

[16] Singh N., Yadav S.S. (2022). Ethnomedicinal uses of Indian spices used for cancer treatment: A treatise on structure-activity relationship and signaling pathways. Curr. Res. Food Sci..

[17] Espíndola K.M.M., Ferreira R.G., Narvaez L.E.M., Silva Rosario A.C.R., da Silva A.H.M., Silva A.G.B., Vieira A.P.O., Monteiro M.C. (2019). Chemical and Pharmacological Aspects of Caffeic Acid and Its Activity in Hepatocarcinoma. Front. Oncol..

[18] Nunes S., Madureira A.R., Campos D., Sarmento B., Gomes A.M., Pintado M., Reis F. (2017). Therapeutic and nutraceutical potential of rosmarinic acid-Cytoprotective properties and pharmacokinetic profile. Crit. Rev. Food Sci. Nutr..

[19] Azuma K., Ippoushi K., Nakayama M., Ito H., Higashio H., Terao J. (2000). Absorption of chlorogenic acid and caffeic acid in rats after oral administration. J. Agric. Food Chem..

[20] Gan R.Y., Li H.B., Sui Z.Q., Corke H. (2018). Absorption, metabolism, anti-cancer effect and molecular targets of epigallocatechin gallate (EGCG): An updated review. Crit. Rev. Food Sci. Nutr..

[21] Antonopoulou I., Sapountzaki E., Rova U., Christakopoulos P. (2021). Ferulic Acid From Plant Biomass: A Phytochemical with Promising Antiviral Properties. Front. Nutr..

[22] Nagaraju G.P., Dariya B., Kasa P., Peela S., El-Rayes B.F. (2022). Epigenetics in hepatocellular carcinoma. Semin. Cancer Biol..

[23] Che Z., Zhou Z., Li S.Q., Gao L., Xiao J., Wong N.K. (2023). ROS/RNS as molecular signatures of chronic liver diseases. Trends Mol. Med..

[24] Qu Z., Sun J., Zhang W., Yu J., Zhuang C. (2020). Transcription factor NRF2 as a promising therapeutic target for Alzheimer’s disease. Free Radic. Biol. Med..

[25] Zhang W., Feng C., Jiang H. (2021). Novel target for treating Alzheimer’s Diseases: Crosstalk between the Nrf2 pathway and autophagy. Ageing Res. Rev..

[26] Sun X., Ou Z., Chen R., Niu X., Chen D., Kang R., Tang D. (2016). Activation of the p62-Keap1-NRF2 pathway protects against ferroptosis in hepatocellular carcinoma cells. Hepatology.

[27] Suzuki T., Motohashi H., Yamamoto M. (2013). Toward clinical application of the Keap1-Nrf2 pathway. Trends Pharmacol. Sci..

[28] Sabitha R., Nishi K., Gunasekaran V.P., Agilan B., David E., Annamalai G., Vinothkumar R., Perumal M., Subbiah L., Ganeshan M. (2020). p-Coumaric acid attenuates alcohol exposed hepatic injury through MAPKs, apoptosis and Nrf2 signaling in experimental models. Chem.-Biol. Interact..

[29] Ding X., Jian T., Li J., Lv H., Tong B., Li J., Meng X., Ren B., Chen J. (2020). Chicoric Acid Ameliorates Nonalcoholic Fatty Liver Disease via the AMPK/Nrf2/NFκB Signaling Pathway and Restores Gut Microbiota in High-Fat-Diet-Fed Mice. Oxidative Med. Cell. Longev..

[30] Shi A., Li T., Zheng Y., Song Y., Wang H., Wang N., Dong L., Shi H. (2021). Chlorogenic Acid Improves NAFLD by Regulating gut Microbiota and GLP-1. Front. Pharmacol..

[31] Shi A., Shi H., Wang Y., Liu X., Cheng Y., Li H., Zhao H., Wang S., Dong L. (2018). Activation of Nrf2 pathway and inhibition of NLRP3 inflammasome activation contribute to the protective effect of chlorogenic acid on acute liver injury. Int. Immunopharmacol..

[32] Li Z., Feng H., Han L., Ding L., Shen B., Tian Y., Zhao L., Jin M., Wang Q., Qin H. (2020). Chicoric acid ameliorate inflammation and oxidative stress in Lipopolysaccharide and d-galactosamine induced acute liver injury. J. Cell. Mol. Med..

[33] Lv H., Hong L., Tian Y., Yin C., Zhu C., Feng H. (2019). Corilagin alleviates acetaminophen-induced hepatotoxicity via enhancing the AMPK/GSK3β-Nrf2 signaling pathway. Cell Commun. Signal..

[34] Chen Z., Ma X., Zhu Y., Zhao Y., Wang J., Li R., Chen C., Wei S., Jiao W., Zhang Y. (2015). Paeoniflorin ameliorates ANIT-induced cholestasis by activating Nrf2 through an PI3K/Akt-dependent pathway in rats. Phytother. Res. PTR.

[35] Fu R., Zhou J., Wang R., Sun R., Feng D., Wang Z., Zhao Y., Lv L., Tian X., Yao J. (2019). Protocatechuic Acid-Mediated miR-219a-5p Activation Inhibits the p66shc Oxidant Pathway to Alleviate Alcoholic Liver Injury. Oxidative Med. Cell. Longev..

[36] Tang F., Wang Z., Zhou J., Yao J. (2023). Salvianolic Acid A Protects against Acetaminophen-Induced Hepatotoxicity via Regulation of the miR-485-3p/SIRT1 Pathway. Antioxidants.

[37] Pang C., Zheng Z., Shi L., Sheng Y., Wei H., Wang Z., Ji L. (2016). Caffeic acid prevents acetaminophen-induced liver injury by activating the Keap1-Nrf2 antioxidative defense system. Free Radic. Biol. Med..

[38] Sanjay S., Girish C., Toi P.C., Bobby Z. (2021). Gallic acid attenuates isoniazid and rifampicin-induced liver injury by improving hepatic redox homeostasis through influence on Nrf2 and NF-κB signalling cascades in Wistar Rats. J. Pharm. Pharmacol..

[39] Luo J., Long Y., Ren G., Zhang Y., Chen J., Huang R., Yang L. (2019). Punicalagin Reversed the Hepatic Injury of Tetrachloromethane by Antioxidation and Enhancement of Autophagy. J. Med. Food.

[40] Al-Khawalde A.A.A., Abukhalil M.H., Jghef M.M., Alfwuaires M.A., Alaryani F.S., Aladaileh S.H., Algefare A.I., Karimulla S., Alasmari F., Aldal’in H.K. (2022). Punicalagin Protects against the Development of Methotrexate-Induced Hepatotoxicity in Mice via Activating Nrf2 Signaling and Decreasing Oxidative Stress, Inflammation, and Cell Death. Int. J. Mol. Sci..

[41] Yu H., Lin L., Zhang Z., Zhang H., Hu H. (2020). Targeting NF-κB pathway for the therapy of diseases: Mechanism and clinical study. Signal Transduct. Target. Ther..

[42] Gu X., Wei M., Hu F., Ouyang H., Huang Z., Lu B., Ji L. (2023). Chlorogenic acid ameliorated non-alcoholic steatohepatitis via alleviating hepatic inflammation initiated by LPS/TLR4/MyD88 signaling pathway. Chem.-Biol. Interact..

[43] Wang Y., Jian S., Li W., Zhao L., Ye G., Shi F., Li L., Zou Y., Song X., Zhao X. (2022). Epigallocatechin-3-gallate ameliorates liver injury secondary to Pseudomonas aeruginosa pneumonia. Int. Immunopharmacol..

[44] Feng D., Guo R., Liao W., Li J., Cao S. (2023). Plantamajoside alleviates acute sepsis-induced organ dysfunction through inhibiting the TRAF6/NF-κB axis. Pharm. Biol..

[45] Wang H., Zhao Y., Zhang Y., Yang T., Zhao S., Sun N., Tan H., Zhang H., Wang C., Fan H. (2022). Effect of Chlorogenic Acid via Upregulating Resolvin D1 Inhibiting the NF-κB Pathway on Chronic Restraint Stress-Induced Liver Inflammation. J. Agric. Food Chem..

[46] Xu K., Lu G., Feng Q., Chen S., Wang Y. (2023). Hepatoprotective effect of protocatechuic acid against type 2 diabetes-induced liver injury. Pharm. Biol..

[47] Gaskell H., Ge X., Nieto N. (2018). High-Mobility Group Box-1 and Liver Disease. Hepatol. Commun..

[48] Lin S.Y., Wang Y.Y., Chen W.Y., Liao S.L., Chou S.T., Yang C.P., Chen C.J. (2017). Hepatoprotective activities of rosmarinic acid against extrahepatic cholestasis in rats. Food Chem. Toxicol..

[49] Xie T., Li K., Gong X., Jiang R., Huang W., Chen X., Tie H., Zhou Q., Wu S., Wan J. (2018). Paeoniflorin protects against liver ischemia/reperfusion injury in mice via inhibiting HMGB1-TLR4 signaling pathway. Phytother. Res. PTR.

[50] Shi H., Shi A., Dong L., Lu X., Wang Y., Zhao J., Dai F., Guo X. (2016). Chlorogenic acid protects against liver fibrosis in vivo and in vitro through inhibition of oxidative stress. Clin. Nutr..

[51] Fu J., Wu H. (2023). Structural Mechanisms of NLRP3 Inflammasome Assembly and Activation. Annu. Rev. Immunol..

[52] Wang D., Gao Q., Wang T., Kan Z., Li X., Hu L., Peng C.Y., Qian F., Wang Y., Granato D. (2020). Green tea polyphenols and epigallocatechin-3-gallate protect against perfluorodecanoic acid induced liver damage and inflammation in mice by inhibiting NLRP3 inflammasome activation. Food Res. Int..

[53] Goodarzi G., Tehrani S.S., Panahi G., Bahramzadeh A., Meshkani R. (2023). Combination therapy of metformin and p-coumaric acid mitigates metabolic dysfunction associated with obesity and nonalcoholic fatty liver disease in high-fat diet obese C57BL/6 mice. J. Nutr. Biochem..

[54] Xin X., Jin Y., Wang X., Cai B., An Z., Hu Y.Y., Feng Q. (2021). A Combination of Geniposide and Chlorogenic Acid Combination Ameliorates Nonalcoholic Steatohepatitis in Mice by Inhibiting Kupffer Cell Activation. BioMed Res. Int..

[55] Wang Y., Huang S., Kong W., Wu C., Zeng T., Xie S., Chen Q., Kuang S., Zheng R., Wang F. (2023). Corilagin alleviates liver fibrosis in zebrafish and mice by repressing IDO1-mediated M2 macrophage repolarization. Phytomedicine.

[56] Friedman S.L., Neuschwander-Tetri B.A., Rinella M., Sanyal A.J. (2018). Mechanisms of NAFLD development and therapeutic strategies. Nat. Med..

[57] Musso G., Cassader M., Gambino R. (2016). Non-alcoholic steatohepatitis: Emerging molecular targets and therapeutic strategies. Nat. Rev. Drug Discov..

[58] Röhrig F., Schulze A. (2016). The multifaceted roles of fatty acid synthesis in cancer. Nat. Rev. Cancer.

[59] Forbes-Hernández T.Y., Giampieri F., Gasparrini M., Afrin S., Mazzoni L., Cordero M.D., Mezzetti B., Quiles J.L., Battino M. (2017). Lipid Accumulation in HepG2 Cells Is Attenuated by Strawberry Extract through AMPK Activation. Nutrients.

[60] Nyandwi J.B., Ko Y.S., Jin H., Yun S.P., Park S.W., Kim H.J. (2021). Rosmarinic Acid Exhibits a Lipid-Lowering Effect by Modulating the Expression of Reverse Cholesterol Transporters and Lipid Metabolism in High-Fat Diet-Fed Mice. Biomolecules.

[61] Ma K., Sheng W., Song X., Song J., Li Y., Huang W., Liu Y. (2023). Chlorogenic Acid from Burdock Roots Ameliorates Oleic Acid-Induced Steatosis in HepG2 Cells through AMPK/ACC/CPT-1 Pathway. Molecules.

[62] Hua Y.Q., Zeng Y., Xu J., Xu X.L. (2021). Naringenin alleviates nonalcoholic steatohepatitis in middle-aged Apoe(-/-)mice: Role of SIRT1. Phytomedicine.

[63] Tu Y., Yang Q., Tang M., Gao L., Wang Y., Wang J., Liu Z., Li X., Mao L., Jia R.Z. (2024). TBC1D23 mediates Golgi-specific LKB1 signaling. Nat. Commun..

[64] Li S., Qian Q., Ying N., Lai J., Feng L., Zheng S., Jiang F., Song Q., Chai H., Dou X. (2020). Activation of the AMPK-SIRT1 pathway contributes to protective effects of Salvianolic acid A against lipotoxicity in hepatocytes and NAFLD in mice. Front. Pharmacol..

[65] Yu M.H., Hung T.W., Wang C.C., Wu S.W., Yang T.W., Yang C.Y., Tseng T.H., Wang C.J. (2021). Neochlorogenic Acid Attenuates Hepatic Lipid Accumulation and Inflammation via Regulating miR-34a In Vitro. Int. J. Mol. Sci..

[66] Guo D., Bell E.H., Mischel P., Chakravarti A. (2014). Targeting SREBP-1-driven lipid metabolism to treat cancer. Curr. Pharm. Des..

[67] Kohjima M., Higuchi N., Kato M., Kotoh K., Yoshimoto T., Fujino T., Yada M., Yada R., Harada N., Enjoji M. (2008). SREBP-1c, regulated by the insulin and AMPK signaling pathways, plays a role in nonalcoholic fatty liver disease. Int. J. Mol. Med..

[68] Jensen-Urstad A.P., Semenkovich C.F. (2012). Fatty acid synthase and liver triglyceride metabolism: Housekeeper or messenger?. Biochim. Biophys. Acta.

[69] Mohammadi M., Abbasalipourkabir R., Ziamajidi N. (2023). Fish oil and chicoric acid combination protects better against palmitate-induced lipid accumulation via regulating AMPK-mediated SREBP-1/FAS and PPARα/UCP2 pathways. Arch. Physiol. Biochem..

[70] Zhang Y., Yin R., Lang J., Fu Y., Yang L., Zhao D. (2022). Epigallocatechin-3-gallate ameliorates hepatic damages by relieve FGF21 resistance and promotion of FGF21-AMPK pathway in mice fed a high fat diet. Diabetol. Metab. Syndr..

[71] Kim M., Yoo G., Randy A., Kim H.S., Nho C.W. (2017). Chicoric acid attenuate a nonalcoholic steatohepatitis by inhibiting key regulators of lipid metabolism, fibrosis, oxidation, and inflammation in mice with methionine and choline deficiency. Mol. Nutr. Food Res..

[72] Zamani-Garmsiri F., Ghasempour G., Aliabadi M., Hashemnia S.M.R., Emamgholipour S., Meshkani R. (2021). Combination of metformin and chlorogenic acid attenuates hepatic steatosis and inflammation in high-fat diet fed mice. IUBMB Life.

[73] Bougarne N., Weyers B., Desmet S.J., Deckers J., Ray D.W., Staels B., De Bosscher K. (2018). Molecular Actions of PPARα in Lipid Metabolism and Inflammation. Endocr. Rev..

[74] Guo C., Zhang X., Yu Y., Wu Y., Xie L., Chang C. (2022). Lonicerae Japonicae Flos extract and chlorogenic acid attenuates high-fat-diet- induced prediabetes via CTRPs-AdipoRs-AMPK/PPARα axes. Front. Nutr..

[75] Yuan Z., Lu X., Lei F., Sun H., Jiang J., Xing D., Du L. (2023). Novel Effect of p-Coumaric Acid on Hepatic Lipolysis: Inhibition of Hepatic Lipid-Droplets. Molecules.

[76] Tao Z., Zhang L., Wu T., Fang X., Zhao L. (2021). Echinacoside ameliorates alcohol-induced oxidative stress and hepatic steatosis by affecting SREBP1c/FASN pathway via PPARα. Food Chem. Toxicol..

[77] Cheng Q., Li Y.W., Yang C.F., Zhong Y.J., He H., Zhu F.C., Li L. (2018). Methyl ferulic acid attenuates ethanol-induced hepatic steatosis by regulating AMPK and FoxO1 Pathways in Rats and L-02 cells. Chem.-Biol. Interact..

[78] Ding H., Ge K., Fan C., Liu D., Wu C., Li R., Yan F.J. (2024). Chlorogenic Acid Attenuates Hepatic Steatosis by Suppressing ZFP30. J. Agric. Food Chem..

[79] Luo C., Sun H., Peng J., Gao C., Bao L., Ji R., Zhang C., Zhu W., Jin Y. (2021). Rosmarinic acid exerts an antagonistic effect on nonalcoholic fatty liver disease by regulating the YAP1/TAZ-PPARγ/PGC-1α signaling pathway. Phytother. Res. PTR.

[80] Ding C., Zhao Y., Shi X., Zhang N., Zu G., Li Z., Zhou J., Gao D., Lv L., Tian X. (2016). New insights into salvianolic acid A action: Regulation of the TXNIP/NLRP3 and TXNIP/ChREBP pathways ameliorates HFD-induced NAFLD in rats. Sci. Rep..

[81] Nag S., Manna K., Saha M., Das Saha K. (2020). Tannic acid and vitamin E loaded PLGA nanoparticles ameliorate hepatic injury in a chronic alcoholic liver damage model via EGFR-AKT-STAT3 pathway. Nanomedicine.

[82] Deretic V. (2021). Autophagy in inflammation, infection, and immunometabolism. Immunity.

[83] Mizushima N. (2018). A brief history of autophagy from cell biology to physiology and disease. Nat. Cell Biol..

[84] Itakura E., Kishi C., Inoue K., Mizushima N. (2008). Beclin 1 forms two distinct phosphatidylinositol 3-kinase complexes with mammalian Atg14 and UVRAG. Mol. Biol. Cell.

[85] Zhang Y., Tan X., Cao Y., An X., Chen J., Yang L. (2022). Punicalagin Protects against Diabetic Liver Injury by Upregulating Mitophagy and Antioxidant Enzyme Activities. Nutrients.

[86] Glick D., Barth S., Macleod K.F. (2010). Autophagy: Cellular and molecular mechanisms. J. Pathol..

[87] Yao H.T., Li C.C., Chang C.H. (2019). Epigallocatechin-3-Gallate Reduces Hepatic Oxidative Stress and Lowers CYP-Mediated Bioactivation and Toxicity of Acetaminophen in Rats. Nutrients.

[88] Zhang R., Chu K., Zhao N., Wu J., Ma L., Zhu C., Chen X., Wei G., Liao M. (2019). Corilagin Alleviates Nonalcoholic Fatty Liver Disease in High-Fat Diet-Induced C57BL/6 Mice by Ameliorating Oxidative Stress and Restoring Autophagic Flux. Front. Pharmacol..

[89] Matsuda N., Sato S., Shiba K., Okatsu K., Saisho K., Gautier C.A., Sou Y.S., Saiki S., Kawajiri S., Sato F. (2010). PINK1 stabilized by mitochondrial depolarization recruits Parkin to damaged mitochondria and activates latent Parkin for mitophagy. J. Cell Biol..

[90] Geisler S., Holmström K.M., Skujat D., Fiesel F.C., Rothfuss O.C., Kahle P.J., Springer W. (2010). PINK1/Parkin-mediated mitophagy is dependent on VDAC1 and p62/SQSTM1. Nat. Cell Biol..

[91] Narendra D., Kane L.A., Hauser D.N., Fearnley I.M., Youle R.J. (2010). p62/SQSTM1 is required for Parkin-induced mitochondrial clustering but not mitophagy; VDAC1 is dispensable for both. Autophagy.

[92] Cao Y., Ren G., Zhang Y., Qin H., An X., Long Y., Chen J., Yang L. (2021). A new way for punicalagin to alleviate insulin resistance: Regulating gut microbiota and autophagy. Food Nutr. Res..

[93] Wang J., Lai X., Yuan D., Liu Y., Wang J., Liang Y. (2022). Effects of ferulic acid, a major component of rice bran, on proliferation, apoptosis, and autophagy of HepG2 cells. Food Res. Int..

[94] Zhang Y., Cao Y., Chen J., Qin H., Yang L. (2019). A New Possible Mechanism by Which Punicalagin Protects against Liver Injury Induced by Type 2 Diabetes Mellitus: Upregulation of Autophagy via the Akt/FoxO3a Signaling Pathway. J. Agric. Food Chem..

[95] Wu D., Liu Z., Wang Y., Zhang Q., Li J., Zhong P., Xie Z., Ji A., Li Y. (2021). Epigallocatechin-3-Gallate Alleviates High-Fat Diet-Induced Nonalcoholic Fatty Liver Disease via Inhibition of Apoptosis and Promotion of Autophagy through the ROS/MAPK Signaling Pathway. Oxidative Med. Cell. Longev..

[96] Luo L., Lin J., Chen S., Ni J., Peng H., Shen F., Huang Z. (2024). Rosmarinic acid alleviates toosendanin-induced liver injury through restoration of autophagic flux and lysosomal function by activating JAK2/STAT3/CTSC pathway. J. Ethnopharmacol..

[97] Qin L., Tan J., Lv X., Zhang J. (2023). Vanillic acid alleviates liver fibrosis through inhibiting autophagy in hepatic stellate cells via the MIF/CD74 signaling pathway. Biomed. Pharmacother..

[98] Brown S.B., Bailey K., Savill J. (1997). Actin is cleaved during constitutive apoptosis. Biochem. J..

[99] Wang K.K., Posmantur R., Nath R., McGinnis K., Whitton M., Talanian R.V., Glantz S.B., Morrow J.S. (1998). Simultaneous degradation of alphaII- and betaII-spectrin by caspase 3 (CPP32) in apoptotic cells. J. Biol. Chem..

[100] Kothakota S., Azuma T., Reinhard C., Klippel A., Tang J., Chu K., McGarry T.J., Kirschner M.W., Koths K., Kwiatkowski D.J. (1997). Caspase-3-generated fragment of gelsolin: Effector of morphological change in apoptosis. Science.

[101] Boatright K.M., Salvesen G.S. (2003). Mechanisms of caspase activation. Curr. Opin. Cell Biol..

[102] Marzo I., Brenner C., Zamzami N., Susin S.A., Beutner G., Brdiczka D., Rémy R., Xie Z.H., Reed J.C., Kroemer G. (1998). The permeability transition pore complex: A target for apoptosis regulation by caspases and bcl-2-related proteins. J. Exp. Med..

[103] Green D.R., Kroemer G. (2004). The pathophysiology of mitochondrial cell death. Science.

[104] Zou H., Li Y., Liu X., Wang X. (1999). An APAF-1.cytochrome c multimeric complex is a functional apoptosome that activates procaspase-9. J. Biol. Chem..

[105] Budihardjo I., Oliver H., Lutter M., Luo X., Wang X. (1999). Biochemical pathways of caspase activation during apoptosis. Annu. Rev. Cell Dev. Biol..

[106] Wu J., Zhou F., Fan G., Liu J., Wang Y., Xue X., Lyu X., Lin S., Li X. (2022). Ferulic acid ameliorates acetaminophen-induced acute liver injury by promoting AMPK-mediated protective autophagy. IUBMB Life.

[107] Li Y., Deng X., Hu Q., Chen Y., Zhang W., Qin X., Wei F., Lu X., Ma X., Zeng J. (2024). Paeonia lactiflora Pall. ameliorates acetaminophen-induced oxidative stress and apoptosis via inhibiting the PKC-ERK pathway. J. Ethnopharmacol..

[108] Yang Y., Liu M., Zhao T., Chen Q., Yang Y., Wang S., Zhang J., Deng G., Sun K., Nan Y. (2022). Epigallocatechin-3-gallate Mo nanoparticles (EGM NPs) efficiently treat liver injury by strongly reducing oxidative stress, inflammation and endoplasmic reticulum stress. Front. Pharmacol..

[109] Foroutanfar A., Mehri S., Kamyar M., Tandisehpanah Z., Hosseinzadeh H. (2020). Protective effect of punicalagin, the main polyphenol compound of pomegranate, against acrylamide-induced neurotoxicity and hepatotoxicity in rats. Phytother. Res. PTR.

[110] Li M., Liu P., Xue Y., Liang Y., Shi J., Han X., Zhang J., Chu X., Chu L. (2020). Tannic acid attenuates hepatic oxidative stress, apoptosis and inflammation by activating the Keap1-Nrf2/ARE signaling pathway in arsenic trioxide-toxicated rats. Oncol. Rep..

[111] Wu X., Wang J., Li B., Gong M., Cao C., Song L., Qin L., Wang Y., Zhang Y., Li Y. (2023). Chlorogenic acid, rutin, and quercetin from Lysimachia christinae alleviate triptolide-induced multi-organ injury in vivo by modulating immunity and AKT/mTOR signal pathway to inhibit ferroptosis and apoptosis. Toxicol. Appl. Pharmacol..

[112] Yu Q., Chen S., Tang H., Zhang X., Tao R., Yan Z., Shi J., Guo W., Zhang S. (2021). Veratric acid alleviates liver ischemia/reperfusion injury by activating the Nrf2 signaling pathway. Int. Immunopharmacol..

[113] Zhou H.Q., Liu W., Wang J., Huang Y.Q., Li P.Y., Zhu Y., Wang J.B., Ma X., Li R.S., Wei S.Z. (2017). Paeoniflorin attenuates ANIT-induced cholestasis by inhibiting apoptosis in vivo via mitochondria-dependent pathway. Biomed. Pharmacother..

[114] Higashi T., Friedman S.L., Hoshida Y. (2017). Hepatic stellate cells as key target in liver fibrosis. Adv. Drug Deliv. Rev..

[115] Khomich O., Ivanov A.V., Bartosch B. (2019). Metabolic Hallmarks of Hepatic Stellate Cells in Liver Fibrosis. Cells.

[116] Meng X.M., Nikolic-Paterson D.J., Lan H.Y. (2016). TGF-β: The master regulator of fibrosis. Nat. Rev. Nephrol..

[117] Budi E.H., Schaub J.R., Decaris M., Turner S., Derynck R. (2021). TGF-β as a driver of fibrosis: Physiological roles and therapeutic opportunities. J. Pathol..

[118] Walton K.L., Johnson K.E., Harrison C.A. (2017). Targeting TGF-β Mediated SMAD Signaling for the Prevention of Fibrosis. Front. Pharmacol..

[119] Dooley S., Hamzavi J., Breitkopf K., Wiercinska E., Said H.M., Lorenzen J., Ten Dijke P., Gressner A.M. (2003). Smad7 prevents activation of hepatic stellate cells and liver fibrosis in rats. Gastroenterology.

[120] Yang Y., Sun M., Li W., Liu C., Jiang Z., Gu P., Li J., Wang W., You R., Ba Q. (2021). Rebalancing TGF-β/Smad7 signaling via Compound kushen injection in hepatic stellate cells protects against liver fibrosis and hepatocarcinogenesis. Clin. Transl. Med..

[121] Liu J., Kong D., Qiu J., Xie Y., Lu Z., Zhou C., Liu X., Zhang R., Wang Y. (2019). Praziquantel ameliorates CCl_4_-induced liver fibrosis in mice by inhibiting TGF-β/Smad signalling via up-regulating Smad7 in hepatic stellate cells. Br. J. Pharmacol..

[122] Xu X., Guo Y., Luo X., Shen Z., Sun Z., Shen B., Zhou C., Wang J., Lu J., Zhang Q. (2023). Hydronidone ameliorates liver fibrosis by inhibiting activation of hepatic stellate cells via Smad7-mediated degradation of TGFβRI. Liver Int..

[123] Mu M., Zuo S., Wu R.M., Deng K.S., Lu S., Zhu J.J., Zou G.L., Yang J., Cheng M.L., Zhao X.K. (2018). Ferulic acid attenuates liver fibrosis and hepatic stellate cell activation via inhibition of TGF-β/Smad signaling pathway. Drug Des. Dev. Ther..

[124] Yang F., Luo L., Zhu Z.D., Zhou X., Wang Y., Xue J., Zhang J., Cai X., Chen Z.L., Ma Q. (2017). Chlorogenic Acid Inhibits Liver Fibrosis by Blocking the miR-21-Regulated TGF-β1/Smad7 Signaling Pathway in Vitro and in Vivo. Front. Pharmacol..

[125] Liang J., Chen T., Xu H., Wang T., Gong Q., Li T., Liu X., Wang J., Wang Y., Xiong L. (2024). Echinacoside Exerts Antihepatic Fibrosis Effects in High-Fat Mice Model by Modulating the ACVR2A-Smad Pathway. Mol. Nutr. Food Res..

[126] Cui B., Yang Z., Wang S., Guo M., Li Q., Zhang Q., Bi X. (2021). The protective role of protocatechuic acid against chemically induced liver fibrosis in vitro and in vivo. Die Pharm..

[127] Chen C., Li X., Wang L. (2020). Thymosinβ4 alleviates cholestatic liver fibrosis in mice through downregulating PDGF/PDGFR and TGFβ/Smad pathways. Dig. Liver Dis..

[128] Wang X., Gao Y., Li Y., Huang Y., Zhu Y., Lv W., Wang R., Gou L., Cheng C., Feng Z. (2020). Roseotoxin B alleviates cholestatic liver fibrosis through inhibiting PDGF-B/PDGFR-β pathway in hepatic stellate cells. Cell Death Dis..

[129] Ren H., Li Y., Chen Y., Wang L. (2019). Endostatin attenuates PDGF-BB- or TGF-β1-induced HSCs activation via suppressing RhoA/ROCK1 signal pathways. Drug Des. Dev. Ther..

[130] Liu F., Li S., Chen P., Gu Y., Wang S., Wang L., Chen C., Wang R., Yuan Y. (2023). Salvianolic acid B inhibits hepatic stellate cell activation and liver fibrosis by targeting PDGFRβ. Int. Immunopharmacol..

[131] Lan T., Li C., Yang G., Sun Y., Zhuang L., Ou Y., Li H., Wang G., Kisseleva T., Brenner D. (2018). Sphingosine kinase 1 promotes liver fibrosis by preventing miR-19b-3p-mediated inhibition of CCR2. Hepatology.

[132] Mederacke I., Hsu C.C., Troeger J.S., Huebener P., Mu X., Dapito D.H., Pradere J.P., Schwabe R.F. (2013). Fate tracing reveals hepatic stellate cells as dominant contributors to liver fibrosis independent of its aetiology. Nat. Commun..

[133] Luedde T., Schwabe R.F. (2011). NF-κB in the liver—Linking injury, fibrosis and hepatocellular carcinoma. Nat. Rev. Gastroenterol. Hepatol..

[134] Liu X., Huang K., Zhang R.J., Mei D., Zhang B. (2020). Isochlorogenic Acid A Attenuates the Progression of Liver Fibrosis Through Regulating HMGB1/TLR4/NF-κB Signaling Pathway. Front. Pharmacol..

[135] Miao H., Ouyang H., Guo Q., Wei M., Lu B., Kai G., Ji L. (2022). Chlorogenic acid alleviated liver fibrosis in methionine and choline deficient diet-induced nonalcoholic steatohepatitis in mice and its mechanism. J. Nutr. Biochem..

[136] Leshem A., Liwinski T., Elinav E. (2020). Immune-Microbiota Interplay and Colonization Resistance in Infection. Mol. Cell.

[137] Woodhouse C.A., Patel V.C., Singanayagam A., Shawcross D.L. (2018). Review article: The gut microbiome as a therapeutic target in the pathogenesis and treatment of chronic liver disease. Aliment. Pharmacol. Ther..

[138] Ning K., Lu K., Chen Q., Guo Z., Du X., Riaz F., Feng L., Fu Y., Yin C., Zhang F. (2020). Epigallocatechin Gallate Protects Mice against Methionine-Choline-Deficient-Diet-Induced Nonalcoholic Steatohepatitis by Improving Gut Microbiota To Attenuate Hepatic Injury and Regulate Metabolism. ACS Omega.

[139] Jiang Y., Zhao L., Ma J., Yang Y., Zhang B., Xu J., Dhondrup R., Wong T.W., Zhang D. (2024). Preventive mechanisms of Chinese Tibetan medicine Triphala against nonalcoholic fatty liver disease. Phytomedicine.

[140] Ding Y., Li X., Liu Y., Wang S., Cheng D. (2021). Protection Mechanisms Underlying Oral Administration of Chlorogenic Acid against Cadmium-Induced Hepatorenal Injury Related to Regulating Intestinal Flora Balance. J. Agric. Food Chem..

[141] Mu H.N., Zhou Q., Yang R.Y., Tang W.Q., Li H.X., Wang S.M., Li J., Chen W.X., Dong J. (2021). Caffeic acid prevents non-alcoholic fatty liver disease induced by a high-fat diet through gut microbiota modulation in mice. Food Res. Int..

[142] Zhang K., Yang J., Chen L., He J., Qu D., Zhang Z., Liu Y., Li X., Liu J., Li J. (2023). Gut Microbiota Participates in Polystyrene Microplastics-Induced Hepatic Injuries by Modulating the Gut-Liver Axis. ACS Nano.

[143] Naveed M., Hejazi V., Abbas M., Kamboh A.A., Khan G.J., Shumzaid M., Ahmad F., Babazadeh D., FangFang X., Modarresi-Ghazani F. (2018). Chlorogenic acid (CGA): A pharmacological review and call for further research. Biomed. Pharmacother..

[144] Alam M.A. (2019). Anti-hypertensive Effect of Cereal Antioxidant Ferulic Acid and Its Mechanism of Action. Front. Nutr..

[145] Baby B., Antony P., Vijayan R. (2018). Antioxidant and anticancer properties of berries. Crit. Rev. Food Sci. Nutr..

[146] Noguchi-Shinohara M., Ono K., Hamaguchi T., Iwasa K., Nagai T., Kobayashi S., Nakamura H., Yamada M. (2015). Pharmacokinetics, Safety and Tolerability of Melissa officinalis Extract which Contained Rosmarinic Acid in Healthy Individuals: A Randomized Controlled Trial. PLoS ONE.

[147] Lee D.S., Woo J.Y., Ahn C.B., Je J.Y. (2014). Chitosan-hydroxycinnamic acid conjugates: Preparation, antioxidant and antimicrobial activity. Food Chem..

[148] Park S.Y., Ahn G., Um J.H., Han E.J., Ahn C.B., Yoon N.Y., Je J.Y. (2017). Hepatoprotective effect of chitosan-caffeic acid conjugate against ethanol-treated mice. Exp. Toxicol. Pathol..

[149] Zhou C., Zhang L., Xu Z., Sun T., Gong M., Liu Y., Zhang D. (2023). Self-Propelled Ultrasmall AuNPs-Tannic Acid Hybrid Nanozyme with ROS-Scavenging and Anti-Inflammatory Activity for Drug-Induced Liver Injury Alleviation. Small.

[150] Mansour A., Mohajeri-Tehrani M.R., Samadi M., Qorbani M., Merat S., Adibi H., Poustchi H., Hekmatdoost A. (2021). Effects of supplementation with main coffee components including caffeine and/or chlorogenic acid on hepatic, metabolic, and inflammatory indices in patients with non-alcoholic fatty liver disease and type 2 diabetes: A randomized, double-blind, placebo-controlled, clinical trial. Nutr. J..

[151] Clevers H. (2016). Modeling Development and Disease with Organoids. Cell.

[152] Blanchard Z., Brown E.A., Ghazaryan A., Welm A.L. (2025). PDX models for functional precision oncology and discovery science. Nat. Rev. Cancer.

[153] Grosso G., Stepaniak U., Micek A., Kozela M., Stefler D., Bobak M., Pajak A. (2017). Dietary polyphenol intake and risk of type 2 diabetes in the Polish arm of the Health, Alcohol and Psychosocial factors in Eastern Europe (HAPIEE) study. Br. J. Nutr..

[154] Grosso G., Stepaniak U., Micek A., Kozela M., Stefler D., Bobak M., Pajak A. (2018). Dietary polyphenol intake and risk of hypertension in the Polish arm of the HAPIEE study. Eur. J. Nutr..

[155] Grosso G., Stepaniak U., Micek A., Stefler D., Bobak M., Pająk A. (2017). Dietary polyphenols are inversely associated with metabolic syndrome in Polish adults of the HAPIEE study. Eur. J. Nutr..

[156] Rahimlou M., Baghdadi G., Khodi A., Rahimi Z., Saki N., Banaei Jahromi N., Cheraghian B., Tavasolian R., Hosseini S.A. (2024). Polyphenol consumption and Nonalcoholic fatty liver disease risk in adults. Sci. Rep..

[157] Castellino G., Nikolic D., Magán-Fernández A., Malfa G.A., Chianetta R., Patti A.M., Amato A., Montalto G., Toth P.P., Banach M. (2019). Altilix^®^ Supplement Containing Chlorogenic Acid and Luteolin Improved Hepatic and Cardiometabolic Parameters in Subjects with Metabolic Syndrome: A 6 Month Randomized, Double-Blind, Placebo-Controlled Study. Nutrients.

[158] Rodríguez-Daza M.C., Pulido-Mateos E.C., Lupien-Meilleur J., Guyonnet D., Desjardins Y., Roy D. (2021). Polyphenol-Mediated Gut Microbiota Modulation: Toward Prebiotics and Further. Front. Nutr..

[159] Yu C.M., Wang Y., Ren S.C., Liu Z.L., Zhu C.L., Liu Q., Li H.R., Sun C.Y., Sun X.Y., Xie J. (2023). Caffeic acid modulates activation of neutrophils and attenuates sepsis-induced organ injury by inhibiting 5-LOX/LTB4 pathway. Int. Immunopharmacol..

[160] Ajiboye T.O., Ajala-Lawal R.A., Adeyiga A.B. (2019). Caffeic acid abrogates 1,3-dichloro-2-propanol-induced hepatotoxicity by upregulating nuclear erythroid-related factor 2 and downregulating nuclear factor-kappa B. Hum. Exp. Toxicol..

[161] Esmaeilzadeh M., Heidarian E., Shaghaghi M., Roshanmehr H., Najafi M., Moradi A., Nouri A. (2020). Gallic acid mitigates diclofenac-induced liver toxicity by modulating oxidative stress and suppressing IL-1β gene expression in male rats. Pharm. Biol..

[162] Cai Y., Zhao D., Pan Y., Chen B., Cao Y., Han S., Lian F., Zhang Y., Yan X. (2024). Gallic Acid Attenuates Sepsis-Induced Liver Injury through C/EBPβ-Dependent MAPK Signaling Pathway. Mol. Nutr. Food Res..

[163] Mohamed E.K., Hafez D.M. (2023). Gallic acid and metformin co-administration reduce oxidative stress, apoptosis and inflammation via Fas/caspase-3 and NF-κB signaling pathways in thioacetamide-induced acute hepatic encephalopathy in rats. BMC Complement. Med. Ther..

[164] Adeyanju A.A., Asejeje F.O., Molehin O.R., Owoeye O., Olatoye E.O., Ekpo E.N. (2021). Protective role of protocatechuic acid in carbon tetrachloride-induced oxidative stress via modulation of proinflammatory cytokines levels in brain and liver of Wistar rats. J. Basic Clin. Physiol. Pharmacol..

[165] Abdelrahman R.S., El-Tanbouly G.S. (2022). Protocatechuic acid protects against thioacetamide-induced chronic liver injury and encephalopathy in mice via modulating mTOR, p53 and the IL-6/ IL-17/ IL-23 immunoinflammatory pathway. Toxicol. Appl. Pharmacol..

[166] Habib S.A., Suddek G.M., Abdel Rahim M., Abdelrahman R.S. (2021). The protective effect of protocatechuic acid on hepatotoxicity induced by cisplatin in mice. Life Sci..

[167] Tan J., Hu R., Gong J., Fang C., Li Y., Liu M., He Z., Hou D.X., Zhang H., He J. (2023). Protection against Metabolic Associated Fatty Liver Disease by Protocatechuic Acid. Gut Microbes.

[168] Li N., Du X., Qu T., Ren H., Lu W., Cui X., Hu J., Chen Z., Tao H. (2024). Pharmacodynamic material basis and pharmacological mechanisms of Cortex Mori against diabetes mellitus. J. Ethnopharmacol..

[169] Alamri E.S., El Rabey H.A., Alzahrani O.R., Almutairi F.M., Attia E.S., Bayomy H.M., Albalwi R.A., Rezk S.M. (2022). Enhancement of the Protective Activity of Vanillic Acid against Tetrachloro-Carbon (CCl_4_) Hepatotoxicity in Male Rats by the Synthesis of Silver Nanoparticles (AgNPs). Molecules.

[170] Punvittayagul C., Chariyakornkul A., Jarukamjorn K., Wongpoomchai R. (2021). Protective Role of Vanillic Acid against Diethylnitrosamine- and 1,2-Dimethylhydrazine-Induced Hepatocarcinogenesis in Rats. Molecules.

[171] Gheena S., Ezhilarasan D., Shree Harini K., Rajeshkumar S. (2022). Syringic acid and silymarin concurrent administration inhibits sodium valproate-induced liver injury in rats. Environ. Toxicol..

[172] Luo P., Wang F., Wong N.K., Lv Y., Li X., Li M., Tipoe G.L., So K.F., Xu A., Chen S. (2020). Divergent Roles of Kupffer Cell TLR2/3 Signaling in Alcoholic Liver Disease and the Protective Role of EGCG. Cell. Mol. Gastroenterol. Hepatol..

[173] Mostafa-Hedeab G., Ewaiss Hassan M., Halawa T.F., Ahmed Wani F. (2022). Epigallocatechin gallate ameliorates tetrahydrochloride-induced liver toxicity in rats via inhibition of TGFβ / p-ERK/p-Smad1/2 signaling, antioxidant, anti-inflammatory activity. Saudi Pharm. J. SPJ.

[174] Guan Y., Wu Q., Li M., Chen D., Su J., Zuo L., Zhu B., Li Y. (2023). Epigallocatechin-3-gallate Induced HepG2 Cells Apoptosis through ROSmediated AKT /JNK and p53 Signaling Pathway. Curr. Cancer Drug Targets.

[175] Fatima S., Suhail N., Alrashed M., Wasi S., Aljaser F.S., AlSubki R.A., Alsharidah A.S., Banu N. (2021). Epigallocatechin gallate and coenzyme Q10 attenuate cisplatin-induced hepatotoxicity in rats via targeting mitochondrial stress and apoptosis. J. Biochem. Mol. Toxicol..

[176] Dey P., Olmstead B.D., Sasaki G.Y., Vodovotz Y., Yu Z., Bruno R.S. (2020). Epigallocatechin gallate but not catechin prevents nonalcoholic steatohepatitis in mice similar to green tea extract while differentially affecting the gut microbiota. J. Nutr. Biochem..

[177] Yang C., Wu A., Tan L., Tang D., Chen W., Lai X., Gu K., Chen J., Chen D., Tang Q. (2023). Epigallocatechin-3-Gallate Alleviates Liver Oxidative Damage Caused by Iron Overload in Mice through Inhibiting Ferroptosis. Nutrients.

[178] Yuan W., Li S., Yang Y.N., Gao H., Liu C. (2023). Epigallocatechin-3-gallate ameliorates inflammatory injury caused by sepsis by regulating the lncRNA PVT1/miR-16-5p/TLR4 axis. Cytokine.

[179] Huang J., Li W., Liao W., Hao Q., Tang D., Wang D., Wang Y., Ge G. (2020). Green tea polyphenol epigallocatechin-3-gallate alleviates nonalcoholic fatty liver disease and ameliorates intestinal immunity in mice fed a high-fat diet. Food Funct..

[180] Kang Q., Tong Y., Gowd V., Wang M., Chen F., Cheng K.W. (2021). Oral administration of EGCG solution equivalent to daily achievable dosages of regular tea drinkers effectively suppresses miR483-3p induced metastasis of hepatocellular carcinoma cells in mice. Food Funct..

[181] Sojoodi M., Wei L., Erstad D.J., Yamada S., Fujii T., Hirschfield H., Kim R.S., Lauwers G.Y., Lanuti M., Hoshida Y. (2020). Epigallocatechin Gallate Induces Hepatic Stellate Cell Senescence and Attenuates Development of Hepatocellular Carcinoma. Cancer Prev. Res..

[182] Tang Y., Cao J., Cai Z., An H., Li Y., Peng Y., Chen N., Luo A., Tao H., Li K. (2020). Epigallocatechin gallate induces chemopreventive effects on rats with diethylnitrosamine-induced liver cancer via inhibition of cell division cycle 25A. Mol. Med. Rep..

[183] Huang C.Y., Chang Y.J., Wei P.L., Hung C.S., Wang W. (2021). Methyl gallate, gallic acid-derived compound, inhibit cell proliferation through increasing ROS production and apoptosis in hepatocellular carcinoma cells. PLoS ONE.

[184] Liao M., Zhang R., Wang Y., Mao Z., Wu J., Guo H., Zhang K., Jing Y., Zhang C., Song H. (2022). Corilagin prevents non-alcoholic fatty liver disease via improving lipid metabolism and glucose homeostasis in high fat diet-fed mice. Front. Nutr..

[185] Yan F., Cheng D., Wang H., Gao M., Zhang J., Cheng H., Wang C., Zhang H., Xiong H. (2021). Corilagin Ameliorates Con A-Induced Hepatic Injury by Restricting M1 Macrophage Polarization. Front. Immunol..

[186] Bogahawaththa S., Kawamura T., Bandaranayake U., Hirakawa T., Yamada G., Ishino H., Hirohashi T., Kawaguchi S.I., Wijesundera K.K., Wijayagunawardane M.P.B. (2023). Identification and mechanistic investigation of ellagitannins from Osbeckia octandra that attenuate liver fibrosis via the TGF-β/SMAD signaling pathway. Biosci. Biotechnol. Biochem..

[187] Feng X.H., Xu H.Y., Wang J.Y., Duan S., Wang Y.C., Ma C.M. (2021). In vivo hepatoprotective activity and the underlying mechanism of chebulinic acid from Terminalia chebula fruit. Phytomedicine.

[188] Jin D., Zhang B., Li Q., Tu J., Zhou B. (2020). Effect of punicalagin on multiple targets in streptozotocin/high-fat diet-induced diabetic mice. Food Funct..

[189] Sánchez-Terrón G., Martínez R., Morcuende D., Caballero V., Estévez M. (2024). Pomegranate supplementation alleviates dyslipidemia and the onset of non-alcoholic fatty liver disease in Wistar rats by shifting microbiota and producing urolithin-like microbial metabolites. Food Funct..

[190] Tan X., Long Y., Zhang R., Zhang Y., You Z., Yang L. (2024). Punicalagin Ameliorates Diabetic Liver Injury by Inhibiting Pyroptosis and Promoting Autophagy via Modulation of the FoxO1/TXNIP Signaling Pathway. Mol. Nutr. Food Res..

[191] Du P., Zhang X., Luo K., Li Y., Fu C., Xiao J., Xiao Q. (2022). Curculigoside mitigates hepatic ischemia/reperfusion-induced oxidative stress, inflammation, and apoptosis via activation of the Nrf-2/HO-1 pathway. Hum. Exp. Toxicol..

[192] Gawish R.A., Samy E.M., Aziz M.M. (2024). Ferulic acid protects against gamma-radiation induced liver injury via regulating JAK/STAT/Nrf2 pathways. Arch. Biochem. Biophys..

[193] Daryagasht M., Moosavi M., Khorsandi L., Azadnasab R., Khodayar M.J. (2023). Hepatoprotective and anti-hyperglycemic effects of ferulic acid in arsenic-exposed mice. Food Chem. Toxicol..

[194] Yang L., Jiang L., Jiang D., Liu B., Jin S. (2019). The protective effects of salvianolic acid A against hepatic ischemia-reperfusion injury via inhibiting expression of toll-like receptor 4 in rats. Arch. Med. Sci. AMS.

[195] Bal S.S., Leishangthem G.D., Sethi R.S., Singh A. (2022). P-coumaric acid ameliorates fipronil induced liver injury in mice through attenuation of structural changes, oxidative stress and inflammation. Pestic. Biochem. Physiol..

[196] Cui K., Zhang L., La X., Wu H., Yang R., Li H., Li Z. (2022). Ferulic Acid and P-Coumaric Acid Synergistically Attenuate Non-Alcoholic Fatty Liver Disease through HDAC1/PPARG-Mediated Free Fatty Acid Uptake. Int. J. Mol. Sci..

[197] Mehdi S., Ahmad F.U., Lodhi A.H., Khurshid U., Khalid A.A., Sidiq S.S., Hussain L., Baig M.S. (2022). Protective Effects of p-CA Against Acute Liver Damage Induced by LPS/D-GalN in Wistar Albino Rats. Drug Des. Dev. Ther..

[198] Jia K., Zhang Y., Luo R., Liu R., Li Y., Wu J., Xie K., Liu J., Li S., Zhou F. (2023). Acteoside ameliorates hepatic ischemia-reperfusion injury via reversing the senescent fate of liver sinusoidal endothelial cells and restoring compromised sinusoidal networks. Int. J. Biol. Sci..

[199] Yao Y., Li R., Liu D., Long L., He N. (2022). Rosmarinic acid alleviates acetaminophen-induced hepatotoxicity by targeting Nrf2 and NEK7-NLRP3 signaling pathway. Ecotoxicol. Environ. Saf..

[200] Guo C., Shangguan Y., Zhang M., Ruan Y., Xue G., Ma J., Yang J., Qiu L. (2020). Rosmarinic acid alleviates ethanol-induced lipid accumulation by repressing fatty acid biosynthesis. Food Funct..

[201] Ding Y., Zhang Z., Yue Z., Ding L., Zhou Y., Huang Z., Huang H. (2019). Rosmarinic Acid Ameliorates H_2_O_2_-Induced Oxidative Stress in L02 Cells Through MAPK and Nrf2 Pathways. Rejuvenation Res..

[202] Wang L., Yang H., Wang C., Shi X., Li K. (2019). Rosmarinic acid inhibits proliferation and invasion of hepatocellular carcinoma cells SMMC 7721 via PI3K/AKT/mTOR signal pathway. Biomed. Pharmacother..

[203] Yu Y., Wu Y., Yan H.Z., Xia Z.R., Wen W., Liu D.Y., Wan L.H. (2021). Rosmarinic acid ameliorates acetaminophen-induced acute liver injury in mice via RACK1/TNF-α mediated antioxidant effect. Pharm. Biol..

[204] Jia B., Shang J., Zeng H., Wang X., Fang M., Xu L., Liu X., Wu K., Gong Z., Yang Q. (2023). Hepatoprotective Effects of Rosmarinic Acid on Ovalbumin-Induced Intestinal Food Allergy Mouse Model. Molecules.

[205] Kim M., Yoo G., Randy A., Son Y.J., Hong C.R., Kim S.M., Nho C.W. (2020). Lemon Balm and Its Constituent, Rosmarinic Acid, Alleviate Liver Damage in an Animal Model of Nonalcoholic Steatohepatitis. Nutrients.

[206] Wang A., Gong Y., Pei Z., Jiang L., Xia L., Wu Y. (2022). Paeoniflorin ameliorates diabetic liver injury by targeting the TXNIP-mediated NLRP3 inflammasome in db/db mice. Int. Immunopharmacol..

[207] Liu T., Zhang N., Kong L., Chu S., Zhang T., Yan G., Ma D., Dai J., Ma Z. (2022). Paeoniflorin alleviates liver injury in hypercholesterolemic rats through the ROCK/AMPK pathway. Front. Pharmacol..

[208] Deng X., Li Y., Li X., Zhang Z., Dai S., Wu H., Zhang F., Hu Q., Chen Y., Zeng J. (2022). Paeoniflorin Protects against Acetaminophen-Induced Liver Injury in Mice via JNK Signaling Pathway. Molecules.

[209] Wang T., Zhou X., Kuang G., Jiang R., Guo X., Wu S., Wan J., Yin L. (2021). Paeoniflorin modulates oxidative stress, inflammation and hepatic stellate cells activation to alleviate CCl_4_-induced hepatic fibrosis by upregulation of heme oxygenase-1 in mice. J. Pharm. Pharmacol..

[210] Lan T., Li P., Zhang S.J., Liu S.Y., Zeng X.X., Chai F., Tong Y.H., Mao Z.J., Wang S.W. (2024). Paeoniflorin promotes PPARγ expression to suppress HSCs activation by inhibiting EZH2-mediated histone H3K27 trimethylation. Phytomedicine.

[211] Gao M., Zhang D., Jiang C., Jin Q., Zhang J. (2023). Paeoniflorin inhibits hepatocellular carcinoma growth by reducing PD-L1 expression. Biomed. Pharmacother..

[212] Pei X., Tang S., Jiang H., Zhang W., Xu G., Zuo Z., Ren Z., Chen C., Shen Y., Li C. (2023). Paeoniflorin recued hepatotoxicity under zinc oxide nanoparticles exposure via regulation on gut-liver axis and reversal of pyroptosis. Sci. Total Environ..

[213] Li L., Wang H., Zhao S., Zhao Y., Chen Y., Zhang J., Wang C., Sun N., Fan H. (2022). Paeoniflorin ameliorates lipopolysaccharide-induced acute liver injury by inhibiting oxidative stress and inflammation via SIRT1/FOXO1a/SOD2 signaling in rats. Phytother. Res. PTR.

[214] Li W., Zhou J., Zhang Y., Zhang J., Li X., Yan Q., Han J., Hu F. (2021). Echinacoside exerts anti-tumor activity via the miR-503-3p/TGF-β1/Smad aixs in liver cancer. Cancer Cell Int..

